# Identification of Potential Allelochemicals From Donor Plants and Their Synergistic Effects on the Metabolome of *Aegilops geniculata*

**DOI:** 10.3389/fpls.2020.01046

**Published:** 2020-08-05

**Authors:** Monica Scognamiglio, Bernd Schneider

**Affiliations:** Research Group Biosynthesis/ NMR, Max Planck Institute for Chemical Ecology, Jena, Germany

**Keywords:** allelochemicals, allelopathy, metabolomics, NMR, plant stress

## Abstract

The need for plants to defend themselves, communicate, and somehow contribute to the social life in their ecosystems has triggered the evolution of an astonishing number of diverse chemicals, some of which involved in plant–plant interactions. In the present study, specific aspects of allelopathy are investigated. A combination of bioassays and metabolomics was used in order to study the chemical interactions occurring between three donor species of Mediterranean area (*Arbutus unedo*, *Medicago minima*, *Myrtus communis*) and a receiving species (*Aegilops geniculata*). The biochemical changes occurring in the receiving plant upon the treatments with the donor extracts were studied. Oxidative stress and altered water balance were found to be the major changes in the receiving plant. Putative allelochemicals synthesized by the donor plants were also identified and it was shown that their activity was enhanced by co-occurring metabolites. This study provides evidence that metabolite mixtures are to be taken into consideration for allelopathic activity. Furthermore, not only it reports the chemicals responsible for the activity in the specific system, but it also shows that the response of the receiving plant to the treatment with extracts from donor plants is comparable to the response to other stresses.

## Introduction

Plants are virtually active parts of ecosystems, in continuous exchange of cues and signals not only with the environment, but also with other organisms, despite their sessileness and apparent passiveness (Mescher and Pearse, 2016). Noteworthy, among the organisms a plant has to interact with, other plants are of utmost importance. An example above all is the competition for resources, although it is known that plant–plant interactions are somehow much more complex, involving diverse chemical signals (Holmgren et al., 1997; Semchenko et al., 2014).

A specific type of plant–plant interaction based on chemical signalling is the phenomenon known as allelopathy (Muller, 1966) that has been proposed as one of the forces shaping plant community (Scognamiglio et al., 2013; Da Silva et al., 2017). In the most simplified picture, it implies the existence of a donor plant that produces and releases chemicals able to influence the growth and performance of a receiving plant.

Examples of well-studied allelochemicals include juglone, sorgoleone, benzoxazinoids and several others (Jose and Gillespie, 1998; Macias et al., 2004; Dayan et al., 2010). Besides these, a bulk of plant natural products have been reported to possess allelopathic potential, but data on their activities are usually based on *in vitro* studies aimed only at determining one or few aspects of this multifaceted phenomenon. As a consequence, allelopathic interactions are often far from being clarified and allelopathy seems to be still a questionable topic.

However, it must be underlined at this point that a large body of evidence shows that plants do use their vast arsenal of chemicals in order to interact with both abiotic and biotic factors, including plants (Sudha and Ravishankar, 2002; Kessler and Kalske, 2018). It is well documented, for example, that they are able to signal to conspecific the presence of threatens to their survival, like it happens in case of herbivory (Kuhlisch and Pohnert, 2015). It has been postulated that plants should also be able to perceive signals emitted by neighbors (Caruso and Parachnowitsch, 2016). Plant volatiles, for example, have been proven to be involved in signaling (Caruso and Parachnowitsch, 2016). Finally, plants also use chemical signals in order to regulate their own responses to external stimuli (Karban, 2008).

The ability of plants to emit and receive signals is a crucial point supporting the possibility that they can influence neighbors’ performance through chemicals. However, conclusive comprehension of an allelopathic interaction is not a trivial issue, as the biological activities are governed by a number of different factors: individual chemical structure, antagonistic, additive, sequential or synergistic effects, not to mention the metabolic fate of the emitted allelochemicals (Scognamiglio et al., 2013). Furthermore, not only the actual modes of action are rarely understood, but often any information about the biochemical changes in the receiving plant is missing.

Notwithstanding the above-mentioned limitations, the study of allelopathy is very important. On the one hand, allelochemicals could be suggested as possible eco-friendly herbicides. On the other hand, and above all, the phenomenon of allelopathy should not be overlooked when it comes to study plant community structure and dynamics (Scognamiglio et al., 2013). From the ecological standpoint, the effects could range from extreme changes, like the cases of creation of pure stands by the allelopathic plant (Bell and Muller, 1973), to very slow changes in the distribution patterns of some plant species within a community (Chaves et al., 1997). In the latter case, the effects might be due to marginal, but persistent emission of allelochemicals.

One of the ecosystems in which allelopathy seems to play an important role is the Mediterranean one (Bousquet-Melou et al., 2005). Plants of this area are adapted to the very specific and peculiar pedoclimatic conditions, under which a diverse array of specialized metabolites has evolved; some of these metabolites might be implied in the allelopathic phenomenon. Allelopathic interaction as well as active species within one of these plant communities were evidenced in a previous study (Scognamiglio et al., 2014), where only a general overview of the possible candidates for the biological activity was given.

Three out of this set of plant species were chosen as objects of the present in-depth study, which aimed at answering two main questions i) what are the effects induced in the receiving plant by the donor extracts? ii) Is it possible to identify the hypothetical allelochemicals (or mixture of active compounds)?

The experimental approach, based on a method previously standardized (D’abrosca et al., 2013), was hence directed to study the allelopathic phenomenon both from the donor and receiving plant point of view. The main focus was on the biochemical changes induced in the receiving plant and on the identification of active compounds along with possible interactions between different components of the donor extracts.

## Materials and Methods

### Design of Experiment and Plant and Seed Collection

**Figure 1:**
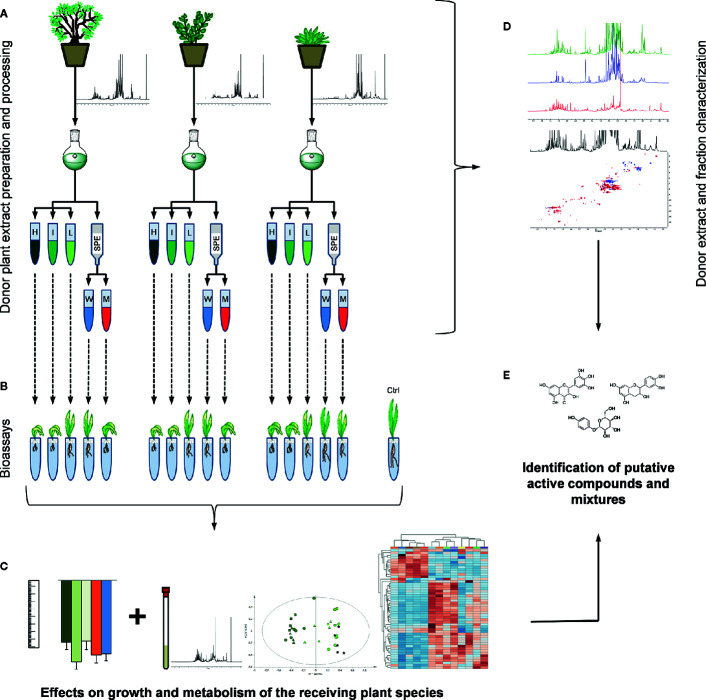
Design of experiment: **(A)** the samples from the three donor plant species were extracted. From each extract three dilutions (H = extract native concentration, I = intermediate concentration, L = low concentration) and two SPE (W = water and M = methanol) fractions were obtained. **(B)** Each one of these preparations was tested in bioassays on a receiving plant species (*A. geniculata*). **(C)** Morphological and metabolomics analyses of the treated receiving plants provided information on the effects on growth and metabolism of the receiving plant. Information deriving from these analyses and from **(D)** an extensive donor extracts and derived fraction characterization allowed **(E)** the identification of putative allelochemicals.

Three donor plant species, namely *Arbutus unedo* L., *Medicago minima* L. and *Myrtus communis* L., respectively belonging to the Ericaceae, Papillionaceae and Myrtaceae family, were tested for their potential of influencing a receiving plant’s (*Aegilops geniculata* Roth., syn. *Triticum vagans* (Jord. & Fourr.) Greuter, Poaceae) growth and performance (**Figures 1A–C**).

Since one of the main aims of this research was to identify the metabolites present in the active extracts, extensive 1D and 2D NMR analyses (**Figure 1D**) were performed on the donor plant extracts and on the fractions obtained by solid phase extraction (SPE). From the combined and comparative analyses of the activity and of the composition of the active fractions, hypotheses on active compounds or mixtures of them could be drawn (**Figure 1E**).

Donor plants were collected at “Castel Volturno” Nature Reserve (40°57’N, 13°33’E; southern Italy), a flat coastal area with a maximum elevation of 9 m above the sea level, characterized by stabilized dunes of alluvial deposits and loose siliceous–calcareous sand and a typically Mediterranean climate (Rutigliano et al., 2004) with landscape dominated by a Mediterranean *macchia* vegetation of mixed shrub and scattered herbaceous community (Esposito et al., 1999). Sampling was carried out on selected plants in March or April 2015, which were the months of maximum production of secondary metabolites for *A. unedo* and *M. communis* (Scognamiglio, unpublished) and the only months in which *M. minima* is found. Leaf samples (obtained by pooling leaves of several biological replicates) of each plant species were harvested and immediately frozen in liquid nitrogen in order to avoid unwanted enzymatic reactions and stored at −80 °C up to the freeze-drying process. Once freeze dried, the samples were powdered in liquid nitrogen and stored at −20 °C.

Voucher specimens for all the plants were deposited at the herbarium of the Department of Environmental, Biological and Pharmaceutical Sciences and Technologies of the Second University of Naples (now University of Campania “Luigi Vanvitelli”). with the following herbarium numbers: *A. unedo* CE000216, *M. minima* CE000229, *M. communis* CE000219.

Seeds of *A. geniculata* were collected in June 2015 at “Castel Volturno” Nature Reserve. Voucher specimens (CE000125) were deposited at the Herbarium of the Department of Environmental, Biological and Pharmaceutical Sciences and Technologies of the Second University of Naples. Yellow caryopses of *A. geniculata* were selected on the basis of their uniformity, by observing them under a binocular microscope Zeiss Stemi 2000 (Oberkochen, Germany) and discarding the undersized and damaged caryopses.

### Extraction and Fractionation

Plant extracts for bioassays were prepared by sonication (20 min) of a mixture of 1.67 g of lyophilized plant material and 50 ml H_2_O/MeOH (1:1). This mixture was then centrifuged at 10,000 rpm (Beckman Avanti J-25, JA-12 rotor) for 10 min. The supernatant was separated from the sediment and evaporated to dryness using rotary evaporator (<40 °C) giving crude extracts (HC). Extract dilutions (1:1, MC and 1:10, LC) were prepared after the extracts were re-solved in the proper amount of water.

Fractions were prepared by SPE of crude extracts using C18 SPE cartridges (Waters, Milford, MA, USA) conditioned with methanol first and water after. The samples were loaded, and the cartridges were eluted first with water to obtain fraction W and then with methanol to obtain fraction M. A washing step with a 5% methanol/water solution was carried out between the two main elution steps. Each fraction was analysed by ^1^H NMR.

### Bioassays

Seeds of the receiving plant were germinated in *Petri* dishes and transferred, after 24 h, to the hydroponic system made up by a tube filled with 5 ml of Hoagland solution (KH_2_PO_4_ 0.50 mM, K_2_HPO_4_ 0.50 mM, K_2_SO_4_ 1.25 mM, MgSO_4_ 2.05 mM, CaCl_2_ 1.00 mM, KNO_3_ 5.00 mM, KCl 25.0 µM, H_3_BO_3_ 12.5 µM, CuSO_4_ 0.25 µM, ZnSO_4_ 1.78 µM, Na_2_MoO_4_ 82 nM, FeCl_3_ 25 µM, Na_2_EDTA 28 µM) and with a 3 mm layer of perlite, for mechanical support. Hoagland solution was added daily. Plants were placed in a Vötsch growth chamber with controlled temperature and relative humidity (27 °C and 60%, respectively), under a photoperiod of 16 h light and 8 h darkness.

Seven days after sowing the plants were treated either with the HC extracts, with the dilutions MC and LC, or the SPE fractions (M and W). Pure compounds arbutin, catechin, gallic, quinic and shikimic acid (Sigma-Aldrich) were analogously tested. The extracts, dilutions, or pure compounds were dissolved in 5 ml of distilled water, and 500 µl of such a solution was added to each tube to obtain the desired concentration. The Hoagland volume was adjusted to 5 ml. Controls were made up in the same way, adding 500 µl of distilled water to the tubes. The experiments were designed so that the concentration of the compounds in the SPE fraction corresponds to the ones in the extracts. The concentrations of the pure compounds were also chosen accordingly. Each treatment was carried out in ten replicates.

The plants were harvested one week after treatment and root and shoot lengths were measured. Measures of both controls and treated plants were corrected against root and leaf length of 7-day old plants. Plants were immediately frozen, and then lyophilized.

### NMR-Based Metabolomics Analysis

Freeze-dried and powdered plant material (10 mg) was transferred to a microtube. The NMR samples were prepared by mixing the lyophilized plant material with 300 µl of the NMR solvent consisting of a phosphate buffer (90 mM; pH 6.0) in D_2_O (Sigma-Aldrich, St. Louis, MO, USA)-containing 0.01% w/v trimethylsilylpropionic-2,2,3,3-*d*_4_ acid sodium salt (TMSP, Sigma-Aldrich)-and CD_3_OD (Sigma-Aldrich) (1:1). The mixture was vortexed at room temperature for 1 min, ultrasonicated (Bandelin Sonorex RX100) for 20 min, and centrifuged (Eppendorf 5415R F45-24-11 rotor) at 13,000 rpm for 10 min. An aliquot of the supernatant was transferred to an NMR capillary tube and analyzed by NMR (Kim and Verpoorte, 2010).

NMR spectra were recorded on a Bruker Avance III HD 700 NMR spectrometer (operating at 700.13 MHz for ^1^H and 175.75 MHz for ^13^C) (Bruker Biospin, Karlsruhe, Germany) equipped with a microcryoprobe (TCI 1.7 mm). All spectra were measured at 300 K. The TMSP signal was used for referencing the spectra.

A 1D NOESY sequence was used to suppress the residual HDO signal. Each ^1^H NMR spectrum consisted of 256 scans with the following parameters: fid resolution 0.17 Hz, acquisition time (AQ) = 5.87 s, relaxation delay (RD) = 2 s. Free induction decays (FIDs) were Fourier-transformed and the resulting spectra were manually phased and baseline-corrected and calibrated to TMSP at 0.0 ppm, using ^1^H NMR processor (ACDLABS 12.0, Toronto, Canada).

^1^H–^1^H correlated spectroscopy (COSY), total correlation spectroscopy (TOCSY), heteronuclear single quantum coherence (HSQC) and heteronuclear multiple bond correlation (HMBC), heteronuclear 2 bonds correlation (H2BC) and HSQC-TOCSY spectra were acquired on selected samples. Standard pulse sequences from Bruker were used and the parameters were adjusted for each sample.

### Multivariate Data Analysis

^1^H NMR spectra were scaled to total intensity and bucketed, reducing them to integral segments with a width of 0.04 ppm with ACDLABS 12.0 ^1^H NMR processor (ACDLABS). The regions at δ −0.02–0.02, 4.70–4.90 and 3.30–3.34 were excluded from the analysis (by indicating them as dark regions before integration) because of residual TMSP and solvents signals. Principal component analysis (PCA) and partial least square discriminant analysis (PLS-DA) were performed with the SIMCA-P software (version 14.0, Umetrics, Umeå Sweden) with scaling based on Pareto and unit variance methods, respectively. Further data analyses were carried out with Metaboanalyst 4.0 (Chong et al., 2018). In particular, one-way ANOVA test followed by post-hoc analysis (Fisher LSD) was used along with PLS-DA to choose the most significant variables to submit to hierarchical cluster analysis (HCA) and pathway analysis. Then, HCA was performed on the top 50 selected variables using Euclidean distance (dissimilarity) with complete linkage as the agglomeration method. From this analysis heat maps were generated. Finally, the list (KEGG identifiers) of metabolites corresponding to these variables was used for pathway analysis.

### Statistical Analysis

The bioassays were carried out on ten biological replicates per treatments (five in case of the treatments with the pure compounds), while metabolomics analyses were carried out on five replicates. Statistical analyses of the morphological data were performed using Excel 2010 (Microsoft Corporation; Redmond, WA, USA). Student’s *t* test (P <0.001) or ANOVA (Analysis of variance) test (P <0.001) were used to test the significance of the observed changes.

## Results

### Receiving Plant Response: Morphological and Metabolic Changes

**Figure 2:**
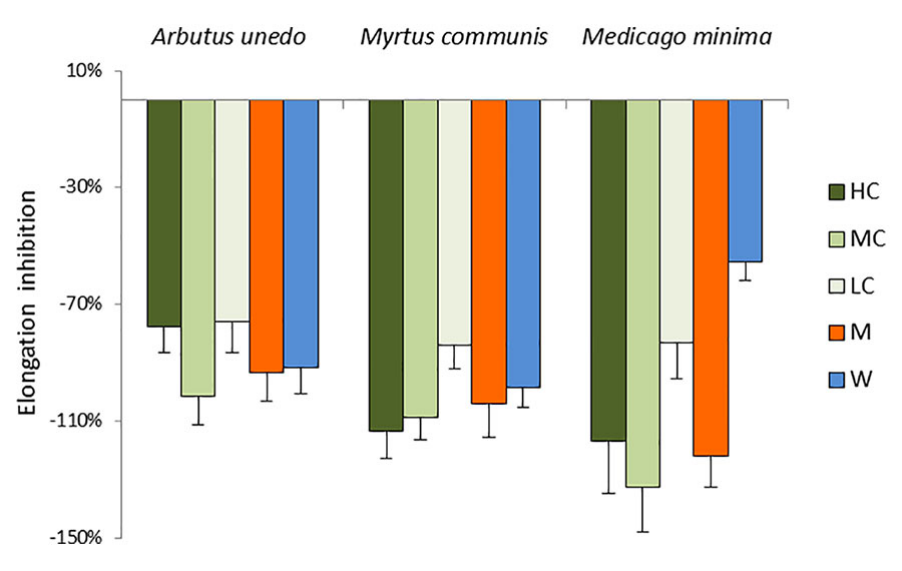
Morphological analysis of *A. geniculata* plants treated with donor plant extracts: inhibition of the leaf growth expressed as % variation from control (±SD; n = 10). Variations from control were significant according to *t* test (P ≤0.001 for all the treatment, with the exception of MW, which was significant for P ≤0.01). HC = High concentration; MC = Medium concentration (1:1); LC = Low concentration (1:10); M = SPE fraction eluted with methanol; W = SPE fraction eluted with water.

The receiving plant, *A. geniculata*, strongly responded to the treatments with the donor extracts (HC) and their dilutions (MC and LC), as well as to the SPE fractions (M and W). The effects were evident both on the leaves and on the roots (**Figure 2**, **Figure S1**, **Table S1**), although the root elongation data were not statistically significant. The three donor extracts induced a significant inhibition of the leaf elongation (**Figure 2**). The inhibition of leaf elongation observed for the extract dilutions (MC and LC) was comparable to the effects observed for the extracts, with the exception of the treatments with *M. minima*, for which the treatment with LC was still significantly different from control, but also significantly different from the HC and MC treatments. The SPE fractions (M and W) were also inhibiting the growth of roots and shoots of the receiving plants, again to a similar degree as the donor extracts, with the exception of the water fraction from *M. minima*, whose effects were milder.

Treatments with the three donor extracts were causing root necrosis, leaf chlorosis and wilting (**Figure S1**). These effects were partially observed already 3 days after the extracts were added to the nutrient solution. Treatments with MC resulted in similar effects than treatments with HC, while for LC milder (for the donor plants *A. unedo* and *M. communis*) or almost no effect (in case of *M. minima*) were detected. The effects caused by the HC were also observed for the treatments with the M fraction of *A. unedo* and *M. communis*, while the analogous fraction of *M. minima* only induced minor symptoms.

The main aim of this study was to elucidate the effects of the donor extracts on the metabolism of *A. geniculata*. Therefore, ^1^H NMR spectra measured from extracts of the receiving plant treated with HC and LC and of the controls were bucketed and the obtained data were analyzed by principal component analysis. Once this analysis was performed, the metabolites corresponding to the buckets of interest were identified based on extensive 1D and 2D NMR analysis and with reference to the previously published *A. geniculata* metabolome (D’abrosca et al., 2013). The samples from plants treated with MC were not included in the metabolome analysis due to the high similarity with the samples from plants treated with HC.

**Figure 3:**
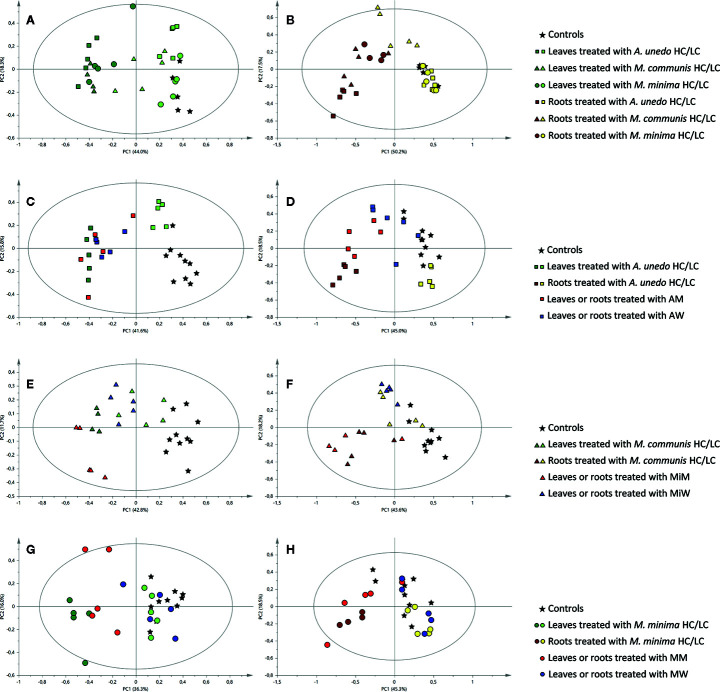
Principal component analysis of ^1^H NMR data of samples obtained from the receiving plant, *A. geniculata*, treated with the donor plant extracts and SPE fractions: score scatter plots of PC1 versus PC2. The ellipses represent the Hotelling T2 with 95% confidence. Different shapes stand for different donor plants and different colors stand for different extracts or fractions, as indicated in the legend. **(A)** leaf samples of *A. geniculata* treated with the three extracts at two different concentrations; **(B)** root samples of *A. geniculata* treated with the three extracts at two different concentrations; **(C)** leaf samples of *A. geniculata* treated with *A. unedo* extracts and SPE fractions; **(D)** root samples of *A. geniculata* treated with *A. unedo* extracts and SPE fractions; **(E)** leaf samples of *A. geniculata* treated with *M. communis* extracts and SPE fractions; **(F)** root samples of *A. geniculata* treated with *M. communis* extracts and SPE fractions; **(G)** leaf samples of *A. geniculata* treated with *M. minima* extracts and SPE fractions; **(H)** root samples of *A. geniculata* treated with *M. minima* extracts and SPE fractions.

From the analysis of the score scatter plot (**Figures 3A, B**), it is clear that the treatments with the HC extracts induced drastic metabolic changes in both the leaves and roots of the receiving plant. Indeed, samples from leaves and roots (indicated by dark green and brown colors, respectively, in **Figure 3**) of *A. geniculata* treated with the highest concentration of the donor extracts were clearly separated from controls along the first principal component.

In particular, the leaves of treated plants divided in two different groups (**Figure 3A**): controls and samples from leaves treated with LC extracts appeared on the positive side of the first principal component axis, whereas samples from leaves treated with HC extracts appeared on the negative side of the same axis. Samples from leaves treated with LC fractions from *A. unedo* and *M. communis* (indicated by squares and triangles, respectively) were also partially separated from controls (asterisk), while the samples from leaves treated with LC from *M. minima* (circles) were not different from controls.

For the leaves treated with HC, a flat gradient of activity was also evident, with *A. unedo* having more drastic effect than the other extracts on the receiving plant metabolism.

From the analysis of the loading plot (**Figure S2**) it was evidenced that the treatment with the most active samples affected both primary and secondary metabolism of the receiving plant. Oblongaroside A (**Figure S3**) and *cis*-aconitic acid showed indirect correlation with PC1, while several amino and organic acids showed a direct correlation (**Figure S2A**). The main variables responsible for the separation along PC2 were those related to sugar signals (increasing along PC2) (**Figure S2B**).

The analysis of the root samples of *A. geniculata* also showed that treatment with the three HC extracts induced more drastic metabolic changes than treatment with LC extracts. Indeed, two groups separated along PC1 could be observed in the score plot: samples treated with LC clustered on the positive side of the first principal component, while on the opposite side of the axis samples treated with HC were observed (**Figure 3B**). Exceptions to this finding were the root samples treated with *M. communis* LC; this group appeared in the middle of the score plot and was separated from controls both along PC1 and along PC2.

PCA of ^1^H NMR data was carried out for samples obtained from treatments of *A. geniculata* with extracts and SPE fractions (M and W) from each donor plant species. The effects of the M and W SPE fractions were compared to the effects of controls and the HC and LC treatments (**Figures 3C–H**).

In case of *A. unedo* (**Figure 3C**), M and W fraction induced comparable changes on the leaf metabolism of *A. geniculata.* Furthermore, these effects were also very similar to those induced by HC. For treatments of the root samples, the activity increased in the order W < M < HC (**Figure 3D**).

In case of samples treated with *M. communis* (**Figure 3E**), both the fractions M and W showed a significant effect on the leaves of the receiving plant. The effects on the root samples treated with *M. communis* were comparable for the HC extract and the M fraction (**Figure 3F**) on one hand and for the LC extract and the W fraction on the other hand. The former cluster was separated from the controls along PC1, while the latter was separated along PC2.

Finally, in case of *M. minima* (**Figures 3G, H**), the effects induced by the W fraction were minimal and comparable to LC on both root and leaf samples, while the response induced by the M fraction was different along PC2, closer, but not completely superimposable to HC for the leaf and root samples.

### Effects of Treatments on the Receiving Plant Metabolism

**Figure 4:**
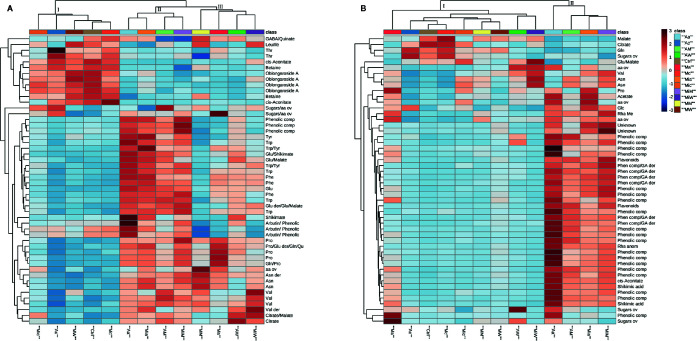
Heat maps of *A. geniculata* leaf **(A)** and root **(B)** data. The analysis was performed using Euclidean distance and average clustering algorithm. *A. geniculata* treated with: Aa = *A. unedo* HC; Ac *A. unedo* LC; AM = *A. unedo* Methanol fraction; AW = *A. unedo* Water fraction; Mia = *M. communis* HC; Mic *M. communis* LC; MiM = *M. communis* Methanol fraction; MiW = *M. communis* Water fraction; Ma = *M. minima* HC; Mc *M. minima* LC; MM = *M. minima* Methanol fraction; MW = *M. minima* Water fraction. Standard abbreviations for amino acids were used. Further abbreviations: aa = amino acids, comp = compound, ov = overlapped.

To further explore the effects of the treatments on the receiving plant metabolism, hierarchical clustering and pathway analyses were carried out. In order to evidence the most significant variables, ANOVA test and PLS-DA (with calculation of VIPs) were performed (**Figures S4** and **S5**, **Tables S2** and **S3**). The top 50 variables identified by one way ANOVA and post hoc analysis were used for heat map generation. From the heat map of the leaf extracts of *A. geniculata* (**Figure 4A**), it was possible to gain information on the metabolic changes induced in the receiving plants, but also once again on the donor plants’ effects.

First of all, the results of the treatments could be assigned to three groups: Group I including the controls, the three LC treatments and the treatment with the W fraction from *M. minima*; group II including the treatments with HC of *A. unedo* and *M. communis* and the respective M fractions, and finally group III made up by the treatments with W fractions of the aforementioned extracts, *M. minima* HC, and its M fraction.

Since group I contained the controls, the other two groups were compared to this one. In case of group II, a decrease of ^1^H NMR signals belonging to oblongaroside A, betaine, *cis*-aconitate and the amino acids threonine, leucine and isoleucine was observed, while an increase of aromatic amino acids and of valine, asparagine, glutamine, glutamic acid and proline was registered. Furthermore, an increase of the organic acids citrate, malate and shikimate was observed. Finally signals in accordance with arbutin and in general more signals in the aromatic region were detected. In group III, the increase in aromatic amino acids and other organic compounds was not as extreme as for group II. Finally, some amino acids derivatives not present in the controls were detected upon treatments (clustering in groups II and III). These might be due to small oligopeptides.

The effects at the root level were less diversified, with only the treatments with *A. unedo* and *M. communis* HC extracts and M fractions significantly separated from the controls (**Figure 4B**). These samples were characterized by a decrease in the amount of the organic acids malate and citrate, variation in the concentration of some aliphatic amino acids and an increase in signals belonging to aromatic metabolites (which was massive in case of *A. unedo* HC treatment).

The metabolic pathway analysis (**Figure S6**) confirmed that the main effects of the active fractions were targeting the amino acid metabolism, TCA cycle and glyoxylate and dicarboxylic acid pathways.

The multivariate data analyses gave also important hints about specific regions of the NMR spectra of the receiving plant extracts that needed visual inspection.

**Figure 5:**
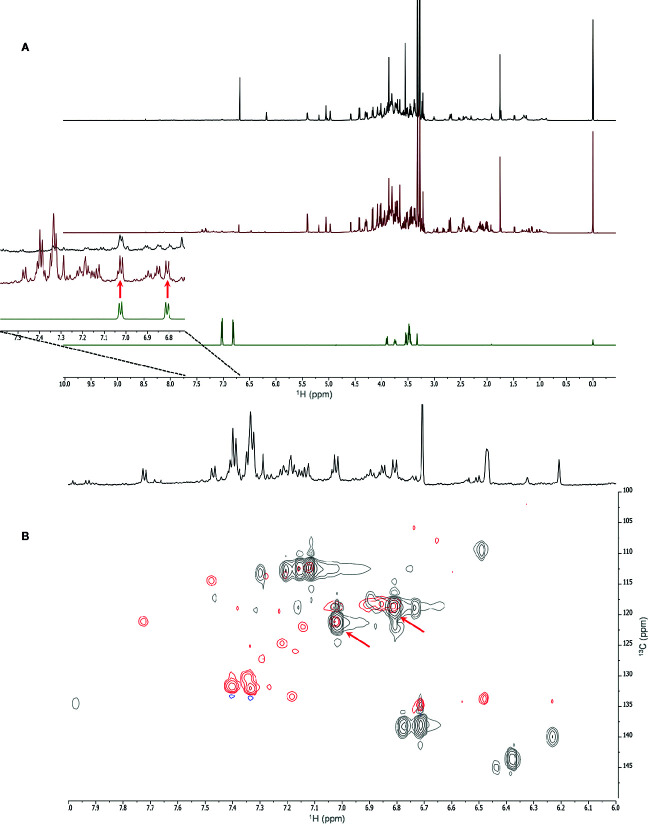
NMR spectra (700 MHz, MeOH-*d*_4_: phosphate buffer in D_2_O 1:1) of *A. geniculata* treated with *A. unedo.* ^1^H-NMR of **(A)** control (black) vs. HC treatment (red) and pure arbutin standard (green); **(B)** Aromatic region of the HSQC spectra of extracts of treated plants (red) vs. water fraction of *A. unedo* (black). The diagnostic signals and correlations of arbutin are indicated by arrows.

The uptake and translocation to the leaf of arbutin was clear in the extracts of *A. geniculata* treated with *A. unedo* (**Figure 5**) and *M. communis*. The compound was identified thanks to extensive 1D and 2D NMR analysis. Two doublets in the aromatic region (δ_H_ 6.81 and 7.02, J = 8.4 Hz) showed HSQC correlations with the respective carbon signals (δ_C_ 118.6 and 121.1). The proton signal at δ_H_ 6.81 showed HMBC correlations with the carbon signals at δ_C_ 121.1 and 153.5. The proton signal at δ_H_ 7.02 showed HMBC correlations with the carbon signals at δ_C_ 118.6 and 154.6. These data were in accordance with a *para*-substituted aromatic system. The carbon resonating at δ_C_ 153.5 also showed long range correlations with a proton at δ_H_ 4.87, in turn showing HSQC correlation with a carbon at δ_C_ 104.6. These signals were assignable to the proton and the carbon atom of the anomeric centre of the sugar moiety. Based on the HSCQ-TOCSY experiment, it was possible to identify the entire hexose spin system and confirm the identity of the glucose unit of arbutin. Other compounds from *A. unedo* and *M. communis* donor extracts were not translocated to the leaves or were below the limit of detection by NMR (**Figure S7**).

The NMR analyses of the root extracts of the receiving plants after treatments with these two donor extracts (*A. unedo* and *M. communis*) showed signals belonging to several gallic acid derivatives (**Figure S8**) absent in the controls. Several diagnostic singlets in the area between 6.9 and 7.5 ppm, all showed a similar pattern of HMBC correlations as those described for gallic acid derivatives in the spectra of donor extracts (**Table 2**). However, differences in the ^1^H NMR chemical shifts between the compounds observed in the root extracts of the receiving plant and the ones in the donor extract suggested structural variants. Therefore, these compounds are derived either from *de novo* biosynthesis induced by stress conditions or by chemical modification of the metabolites possibly taken up by the receiving plant. In case of the treatments with *M. minima*, no compound deriving from the donor extract was detected in the extracts of the receiving plants, neither in the roots nor in the leaves (**Figures S7** and **S8**).

### Characterization of Donor Extracts and Putative Allelochemicals

**Figure 6:**
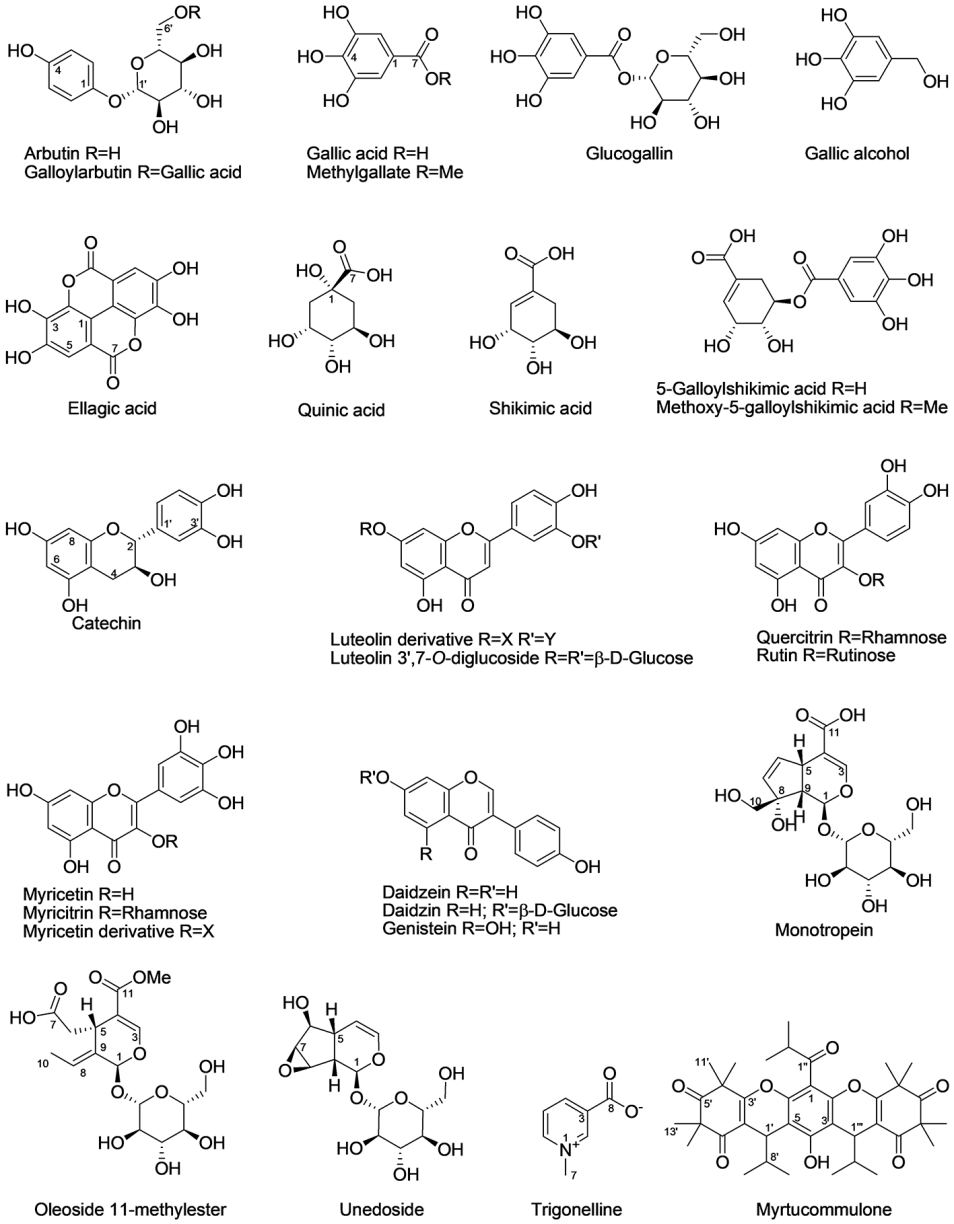
Chemical structures of the main specialized metabolites detected in the donor extracts. The confidence level for the identification of each compound is reported in **Table 2**.

**Figure 7:**
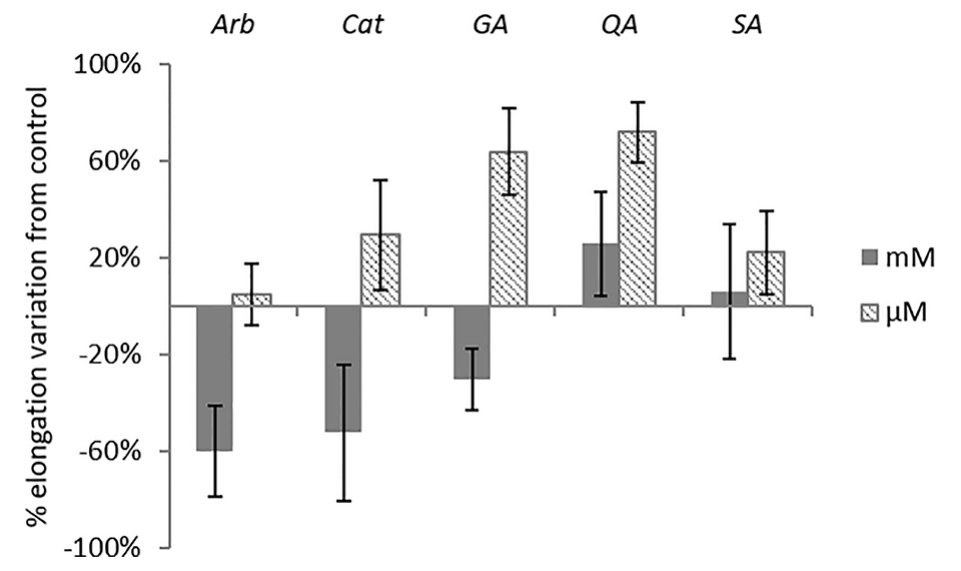
Morphological analysis of *A. geniculata* plants treated with selected pure compounds: inhibition of the leaf growth expressed as % variation from control (±SE; n = 5). Only Arb mM and Ga μM variations were significant according to *t* test (P ≤0.05). Arb = arbutin, Cat = catechin, Ga = gallic acid, QA = quinic acid, SA = shikimic acid.

**Figure 8:**
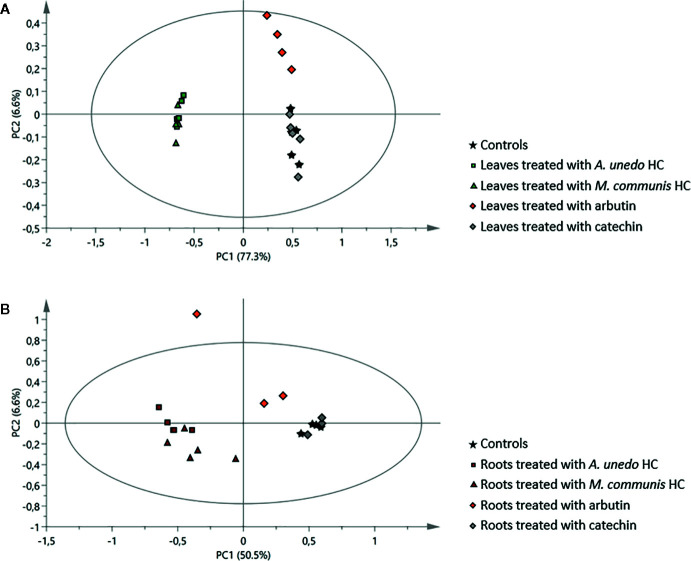
Principal component analysis of ^1^H NMR data of samples obtained from the receiving plant, *A. geniculata*, treated with the *A. unedo* and *M. communis* donor plant extracts (HC) and with pure arbutin and catechin: score scatter plots of PC1 versus PC2. **(A)** effects on leaves, **(B)** effects on roots. The ellipses represent the Hotelling T2 with 95% confidence.

**Figure 9:**
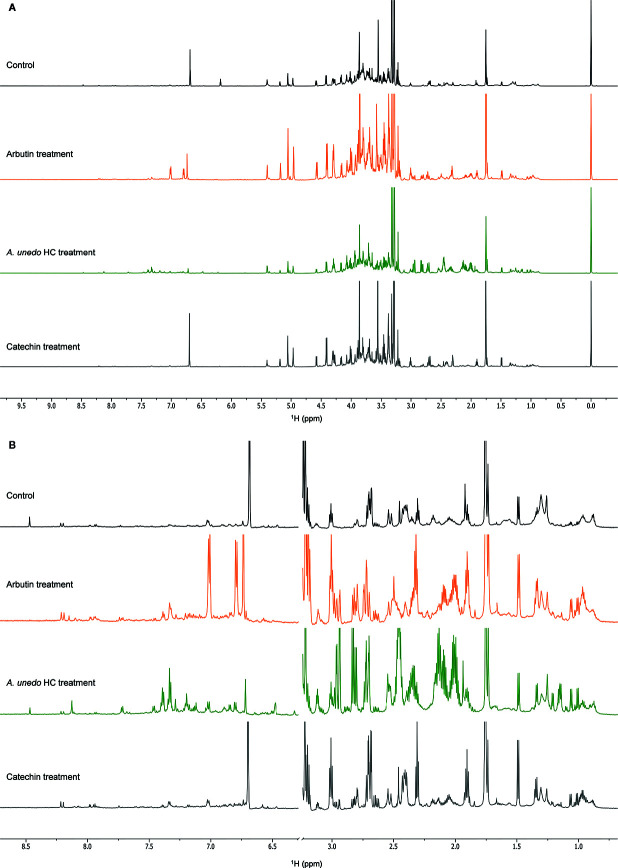
NMR spectra (700 MHz, MeOH-*d*_4_: phosphate buffer in D_2_O 1:1) of *A. geniculata* **(A)** control (black) vs. treatment with pure arbutin (orange), *A. unedo* extract (green), pure catechin (grey) **(B)** details of the aromatic and the aliphatic regions.

The extensive 1D and 2D NMR analysis of the donor extracts and SPE fractions allowed determining their composition (**Figure 6**, **Tables 1** and **2**). By crossing these data with the results of the bioassays, it was possible to abstract putative active compounds (i.e. metabolites that were detected in different fractions triggering the same response). In order to validate this, tests with the pure compounds were performed. Receiving plants were therefore treated with pure arbutin, catechin, gallic acid, quinic acid and shikimic acid at µM and mM concentration. Arbutin and catechin affected plant growth in a negative manner when applied at mM concentration (**Figure 7**). The three acids did not inhibit the growth of the treated plants but instead showed a slight stimulatory activity at the lowest concentration tested (**Figure 7**). The effects at the metabolic level were lower than that of the extracts, even for the active compounds (**Figures 7** and **8**). Aliphatic amino acids and organic acids were the main metabolites affected, but to a lower extent compared to the treatments with the whole extracts and SPE fractions (**Figure 9**). Arbutin was detected in the roots and in the leaves of the treated receiving plant (**Figure 9**), analogously to what observed with the treatments with the entire extracts.

**Table 1:** Metabolites in extracts and SPE fractions (W: water, M: methanol) of donor plants.

	Extracts and SPE fractions					
	*Arbutus unedo*		*Myrtus communis*		*Medicago minima*	
	M	W	M	W	M	W
**Compounds**						
Arbutin		x		x		
Ellagic acid			x			
Gallic acid		x		x		
Gallic acid derivative 1		x				
Gallic acid derivative 2			x			
Gallic acid derivative 3			x			
Gallic alcohol		x				
Glucogallin	x		x			
Methylgallate	x					
Phenolic compound					x	
						
Quinic acid		x		x		
Shikimic acid		x		x		
5-Galloyl shikimic acid		x		x		
Methoxy-5-galloyl shikimic acid	x					
						
Catechin	x		x			
Luteolin derivative					x	
Luteolin-3’,7-*O*-diglucoside					x	
Myricetin			x			
Myricitrin	x		x			
Myricetin derivative			x			
Quercitrin	x		x			
Rutin	x					
Daidzein					x	
Daidzin					x	
Genistein					x	
Monotropein		x				
Oleoside-11-methyl ester	x					
Unedoside		x				
Iridoid x		x				
Iridoid y		x				
Trigonelline						x
Myrtucommulone			x			
Compound x			x			
Alanine				x		x
Asparagine						x
GABA						x
Glutamic acid						x
Glutamine						x
Hydroxylysine						x
Proline						x
Threonine						x
Valine						x
Betaine						x
Choline						x
Glucose		x		x		x
Sucrose		x		x		x
Acetic acid						x
Citric acid						x
Formic acid						x
Isobutyric acid						x
Malic acid						x
Succinic acid				x		x
Fatty acids	x				x	

NMR data for specialized metabolites are reported in **Table 2**, structures in **Figure 6**.

**Table 2:** NMR data (700 MHz, MeOH-d*_4_*: phosphate buffer in D_2_O 1:1).

	^1^H	^13^C (HSQC)	H2BC	HMBC	COSY
**Arbutin (1)**					
1	–	153.5			
2/6	6.81 d (J = 8.4)	118.6	C3/C5	C1, C3	H3/H5
3/5	7.02 d (J = 8.4)	121.1	C2/C6	C2, C4	H2/H6
4	–	154.6			
Glc 1’	4.87 ov	104.6	C2′	C1	
2′		75.9			
6′		64.0			
**Gallic acid (1)**					
1	–	123.7			
2/6	7.11s	112.3		C1, C3, C4, C5, C6, C7	
3/5	–	147.6			
4	–	141.3			
7	–	170.0			
**Gallic acid derivative 1 (3)**					
1	–	123.7			
2/6	7.30s	112.9		C1, C3, C4, C5, C6, C7	
3/5	–	147.6			
4	–	141.1			
7	–	169.2			
**Gallic acid derivative 2 (3)**					
2/6	7.24s	112.2		C1, C3, C4, C5, C6, C7	
3/5	–	147.6			
4	–	141.7			
7	–	170.6			
**Gallic acid derivative 3 (3)**					
2/6	7.18s	112.4		C1, C3, C4, C5, C6, C7	
3/5	–	147.6			
4	–	141.7			
7	–	170.3			
**Gallic alcohol (1)**					
1	–	135.4			
2/6	6.49s	109.4		C1, C3, C4, C5, C6, C7	
3/5	–	148.0			
4	–	135.4			
7	4.65 ov	83.6		C2	
**Galloylarbutin (3)**					
2/6	6.80 ov	118.8	C3/C5		H3/H5
3/5	7.02 ov	121.3	C2/C6		H2/H6
Glc 1	4.87 ov	104.6			
2′/6′	7.22 s	112.8			
**Glucogallin (1)**					
1Glc	5.68 ov	97.6		C7′	
1′	–	121.6			
2′/6′	7.21 s	112.8		C1′, C3′, C4′, C5′, C6′, C7′	
3′/5′	–	148.4			
4′	–	142.6			
7′	–	169.0			
**Methyl gallate (1)**					
1	–	123.0			
2/6	7.11s	112.3		C1, C3, C4, C5, C6, C7	
3/5	–	147.8			
4	–	141.6			
7	–	171.9			
OMe	3.72s	54.2		C7	
**Ellagic acid (2)**					
1	–	112.2			
3	–	139.5			
4	–	147.8			
5	7.31 s	111.8		C1, C3, C4, C7	
7	–	160.4			
**Phenolic compound (3)**					
2/6	7.31 ov	131.5			H3/5
3/5	6.83 ov	118.6			H2/6
**Quinic acid (1)**					
1	–	79.1			
2	2.06 m 1.96 m	40.2	C3	C1, C3, C6, C7	H3
3	4.14 ov	73.6	C2, C4	C1, C2, C4	H2, H4
4	3.54 ov	77.9	C5	C6	H3, H5
5	4.00 ov	70.0	C4, C6	C1, C6	H4, H6
6	2.06 m 1.88 m	43.3	C5	C1, C2, C3, C7	H5
7	–	182.4			
**5-Galloyl shikimic acid (1)**					
1	–	134.1			
2	6.77 s	138.2	C3	C4, C6, C7	H3
3	4.52 ov	68.7	C2,C4	C1, C2, C5	H2
4	4.04 ov	71.3		C2, C6	H5
5	5.31 ov	73.3	C4	C1, C4, C7′	H4, H6
6	2.40 m 2.89 m	31.2	C5	C1, C2, C4, C5	H5
7	–	173.6			
1′	–	122.9			
2′/6′	7.15 s	112.7		C1′, C3′, C4′, C5′, C6′, C7′	
3′/5′	–	147.6			
4′	–	141.3			
7′	–	169.4			
**Shikimic acid (1)**					
1	–	134.5			
2	6.71 brs	137.9	C3	C3, C5, C6, C7	H3
3	4.43 ov	68.7	C2	C1, C2, C4, C5	H2
4	3.72 ov	75.0	C3	C2, C6	H5
5	4.02 ov	69.4		C1	H4
6	2.21ov 2.76 ov	34.3	C5	C1, C2, C4, C5	H5
7	–	174.2			
**Catechin (1)**					
2	4.68 ov	83.9		C3, C4, C8a, C1′, C2′, C6′	
3	4.13 ov	70.0			
4	2.53 dd (J = 16.8, 5.6) 2.83 dd (= 16.8, 8.4)	29.6		C2, C3, C8a, C4a C2, C3, C8a, C4a	
6	6.03 brs	98.4		C8a, C4a	
7	–				
8	5.95 brs	97.6		C4a	
8a	–	158.1			
4a	–	102.8			
1′	–	133.6			
2′	6.88 ov	117.5		C2, C3′, C6′	
3′	–	147.7			
4′	–	147.0			
5′	6.80 ov	118.6		C4′	
6′	6.70 ov	122.4		C2, C2′, C3′	
**Luteolin-3′, 7-*O*-diglucoside (1)**					
2	–	167.9			
3	6.70 s	106.0		C2, C4, C4a	
4	–	186.1			
5	–	163.4			
6	6.51 s	103.0		C5, C7, C8, C4a	
7	–	165.8			
8	6.88 s	98.2		C6, C7, C8a, C4a	
8a	–	160.4			
4a	–	108.8			
1′	–	125.2			
2′	7.79 d (J = 2.0)	118.4		C2, C3′, C4′, C6′	
3′	–	148.1			
4′	–	153.6			
5′	7.04 d (J = 8.4)	119.9	C6′	C1′, C3′, C4′	
6′	7.62 dd (J = 8.4; 2.0)	126.2	C5′	C2, C2′, C4′	
Glc-1	5.24 d (J = 7.7)	102.3	C2-Glc	C7	H2-Glc
2	3.61 ov	74.4			
Glc′-1	5.05 d (J = 7.0)	104.9	C2-Glc′	C3′	H2-Glc′
2	3.68 0v	75.5			
**Luteolin derivative (3)**					
2	–	168.5			
3	6.61 s	105.8		C2, C4, C4a	
4	–	185.3			
6	6.45 s	102.7			
8	6.80 s	98.2			
4a	–	109.1			
1′	–	125.2			
3′	–	148.0			
5′	7.05 ov	117.0		C1′, C3′	
6′	7.56 ov	126.2			
**Myricetin (2)**					
2	–	160.6			
6	6.28 brs				
7	–				
8	6.50 brs				
1′	–	123.4			
2′/6′	7.35 s	112.2		C2, C1′, C3′, C4′, C2′/6′	
3′	–	148.0			
4′	–	139.6			
**Myricetin derivative (2)**					
2	–	160.1			
3	–	137.3			
1′	–	121.8			
2′/6′	6.99 s	111.9		C2, C1′, C3′, C4′, C2′/6′	
3′	–	147.2			
4′	–	139.3			
**Myricitrin (1)**					
2	–	161.6			
3	–	137.5			
5	–	164.0			
6	6.30 brs	101.8		C5, C7, C8, C4a	
7	–	167.1			
8	6.48 brs	96.9		C6, C7, C8a, C4a	
8a	–	159.8			
4a	–	107.6			
1′	–	126.3			
2′/6′	6.99 s	111.9		C2, C1′, C3′, C4′, C2′/6′	
3′	–	148.2			
4′	–	139.3			
1Rha	5.26 brs	104.9		C3	
MeRha	0.93 d (J = 6.3)	19.3			
**Quercitrin (1)**					
2	–	161.4			
3	–	137.4			
5	–	163.9			
6	6.30 brs	102.0		C5, C7, C8, C4a	
7	–	167.0			
8	6.48 brs	97.5		C6, C7, C8a, C4a	
8a	–	159.3			
4a	–	107.3			
1′	–	117.3			
2′	7.37 ov	119.1		C2, C3′, C4′, C6′	
3′	–	151.0			
4′	–	147.8			
5′	6.99 ov	118.7		C2, C1′, C4′	H6′
6′	7.32 ov	125.2		C2, C2′, C3′	H5′
1Rha	5.38 ov	104.7		C3	
MeRha	0.92 d (J = 6.4)	19.2			
**Rutin (2)**					
6	6.30 brs	102.0			
8	6.48 brs	97.5			
2′	7.62 ov	119.2			
5′	6.98 ov	118.7			
6′	7.57 ov	125.4			
1Rha	5.12ov	104.6			
MeRha	0.94 d (J = 6.4)	19.2			
1Glc	4.52 ov	104.0			
**Daidzein (4)**					
2	8.21 s				
**Daidzin (4)**					
2	8.18 s				
**Genistein (4)**					
2	8.18 s				
**Monotropein (1)**					
1	5.60 brs	97.1		C3, C5, C1′	
3	7.43 s	153.9		C1, C4, C5, C11	
4	–	113.7			
5	3.60 ov	40.7			H9
6	6.23 d (J = 4.2)	140.0	C5, C7	C5, C7, C8, C9	H7
7	5.70 d (J = 4.2)	134.9	C6	C5, C6, C8, C9	H6
8	–	87.9			
9	2.71 brs	47.2	C1, C5	C1, C4, C5, C8, C10	H5
10	Ov	69.4			
11	–	173.4			
1′Glc	4.74 d (J = 7.8)	101.3			
**Oleoside 11-methyl ester (2)**					
1	5.84 s	97.1		C8, C1-Glc	
3	7.54 s	151.0		C1, C4, C5, C11	
4	–	110.9			
5	3.95 ov	33.3			H6
6	2.68 ov 2.44 dd (J = 12.6; 7.0)	43.0		C4, C5, C7, C9 C4, C5, C7, C9	H5 H5
7	–	176.0			
8	6.06 q (J = 7.0)	127.4		C1, C10	
9	–	131.7			
10	1.59 d (J = 7.0)	15.4		C8, C9	
11	–	171.7			
1′Glc	4.86	102.4			
Me	3.86 s	58.6		C11	
**Unedoside (1)**					
1	4.86 ov	98.6	C9	C5, C8, C9, C1′	H9
3	6.38 d (J = 5.6)	143.7	C4	C1, C4, C5	H4
4	5.13 brs	105.8	C3, C5	C3, C5, C9	H3, H5
5	2.16 ov	39.0	C4, C6, C9	C1, C3, C4, C6, C9	H4, H9
6	4.03 ov	81.0		C4, C5, C7, C8,	
7	3.57 ov	60.0			
8	3.72 ov	57.6			
9	2.53 t (J = 8.4)	44.9	C1, C5	C1, C6, C7, C8	H1, H5
1′Glc	4.78 ov	102.0			
**Iridoid x (3)**					
1	5.53 brs	97.4		C3, C5	
3	7.48 s	153.9		C1, C4, C5, C11	
4	–	113.7			
5	2.86 ov	40.7		C1, C3, C4, C9	
6	4.04 ov	78.1	C5		
8	–	84.0			
9	2.60 brs	46.7	C1, C5	C1, C4	
10	ov	71.4			
11	–	174.6			
**Iridoid y (3)**					
1	5.44 brs	97.9		C3, C5	
3	7.51 s	154.9		C1, C4, C5, C11	
4	–	114.2			
8	–	36.2			
9	2.28 ov	47.6			
11	–	173.6			
**Trigonelline (1)**					
1	–	–			
2	9.15 s	148.8		C6	
3	–	136.5			
4	8.86 m	147.7	C5	C6	H5
5	8.09 m	132.4		C3, C8	H4, H6
6	8.85 m	147.8	C5	C2	H5
7	4.45 s	50.9		C2, C6	
8	–	170.3			
**Myrtucommulone (3)**					
3	–	119.3			
4	–	150.1			
1′	3.81 ov	29.0		C4, C2′	
2′	–	90.0			
4′	–	51.7			
5′	–	220.7			
6′	–	59.4			
7′	–	214.6			
8′		22.0			
9′/10′	1.70 ov			C5, C1′, C8′	
11′	1.14 s	29.6		C2′, C4′, C5′, C12′	
12′	1.16 s	30.0		C2′, C4′, C5′, C11′	
13′	1.31 s	28.0		C5′, C6′, C7′, C14′	
14′	1.22 s	27.1		C5′, C6′, C7′, C13′	
1′′	–	220.5			
2′′		43.9			
3′′/4′′	1.08 ov	20.3		C1′′, C2′′, C3′′/4′′	
1′′′	3.81 ov			C2′′′	
2′′′	–	100.0			
4′′′	–	55.2			
5′′′	–	216.6			
6′′′	–	57.5			
7′′′	–	202.8			
11′′′	1.06 ov	23.1		C2′′′, C4′′′, C12′′′	
12′′′	1.26 ov	17.5		C2′′′, C4′′′, C11′′′	
13′′′	1.34 ov			C5′′′, C6′′′, C7′′′	
**Compound x (4)**					
1	1.03	25.5		C6	
2	1.03	24.9		C6	
3	1.38 1.47			C6 C6	
4	1.91			C6, C12	
5	6.74			C8, C9, C10, C11, C12	
6	–	82.7			
7	–	112.0			
8	–	118.1			
9	–	138.9			
10	–	141.2			
11	–	147.3			
12	–	170.3			

Chemical shifts are reported in parts per million (ppm) in the scale relative to TMSP; coupling constants (J) are reported in Hz. The confidence level for the identification of each metabolite is indicated in parentheses after the compound name by a number (1–4, in accordance with the rules reported in **Table S4** and **Methods S1**).

## Discussion

### Donor Plants Negatively Affect the Receiving Plant Growth and Performance by Interfering With the Water Status and Inducing Oxidative Stress

In this study, the effect of extracts of three donor species (*A. unedo*, *M. communis*, and *M. minima*) on a receiving plant (*A. geniculata*) was tested. Field observations and previous studies (Scognamiglio et al., 2014) showed that the donor plants have allelopathic potential. This was herewith confirmed by their effects on growth, performance and metabolism of *A. geniculata* (**Figures 2**–**4**, **Figure S1**, **Table S1**). In order to understand the changes in plant metabolism and physiology, metabolomics was performed on the treated receiving plants.

The lowest concentration of the donor plant extracts did not significantly affect the metabolism of the receiving plant (**Figures 3** and **4**), although significant effects on the plant growth were detected (**Figure 2**). Probably, upon the initial perturbation caused by the allelochemicals, the receiving plant has to reallocate resources to defend itself (Ghatak et al., 2018), rather than investing in growth. Hence the overall growth rate is impaired. Once the challenging effects of allelochemicals has ceased, the plant is able to re-adjust its metabolic activity; therefore, the effects are not seen at our harvesting time. At this point, it has to be underlined that the approach herewith used only allows us to take a snapshot of the plant metabolism at a specific stage, corresponding to the sampling time that was chosen based on method standardization (D’abrosca et al., 2013). Further studies using time course experiments could help shedding light on faster effects at metabolic level that are masked in the experiments herewith discussed.

The treatments with HC extracts and the M fractions deriving from *A. unedo* and *M. communis* were inducing an increase in the concentration of many amino acids (**Figure 4**). Amino acid levels changes in stressed plants are connected to different mechanisms including osmotic adjustment (Kaplan et al., 2004), intracellular pH regulation, and detoxification of ROS (Thomason et al., 2018).

In particular, the concentrations of the aromatic amino acids and their precursor, shikimic acid, were increased several folds compared to the control. Although in principle this could be caused either by upstream (e.g., increased biosynthesis rate) or downstream (e.g., decreased degradation rate, protein degradation) processes, it has been suggested that the stress induced accumulation of these compounds is actually due to their *de-novo* biosynthesis (Galili et al., 2016; Hildebrandt, 2018). This biosynthesis upregulation is explained by their role as precursors for many specialized metabolites involved in stress protection (Galili et al., 2016). Although we did not observe accumulation of metabolites deriving from these amino acids in the leaves of the receiving plant, we cannot exclude that they are used for the biosynthesis of compounds that were not analysed here, like volatiles or lignin. On the other hand, in the roots, we did observe accumulation of phenolics, in accordance with the above-mentioned hypothesis.

n addition to aromatic amino acids, other amino acids involved in stress response were upregulated in the leaves. Among these, proline, an important osmolite also involved in the protection against ROS (Nakabayashi and Saito, 2015), was upregulated. These aliphatic amino acids were accumulated also upon treatments with the water fraction of *A. unedo* and *M. communis*.

A pathway that was affected by all of the HC, M and W treatments (with the only exception of the W fraction of *M. minima*) was the TCA cycle: decrease of *cis*-aconitate and increase of malate and citrate were observed in the leaves (**Figure 4A**). The decrease of *cis-*aconitate might be due to the inhibition of cis-aconitase that has been shown to be extremely sensitive to oxidative stress (Baxter et al., 2007). The increase of citrate and malate could derive from fuelling of TCA cycle by other pathways. In the root samples of plants treated with the most active fractions, the opposite was observed (**Figure 4B**), which could be a sign of an accelerated flow through the cycle. The effects on central metabolism, so far discussed, might also indirectly cause the downregulation of the MEP pathway, suggested by the decrease in the level of the hemiterpene oblongaroside A (**Figure 4A**).

Taken together, our observations were answering the first question underlying the present study: what are the effects induced in the receiving plant by the donor extracts?

The accumulation of osmolites, in particular proline, and the observation of lower uptake of the Hoagland solution for the treatments of *A. unedo* and *M. communis* on *A. geniculata* were compatible with an imbalance in water status. The perturbation of TCA cycle and glyoxylate pathway, along with the accumulation of proline and the increased levels of several amino acids suggested the oxidative stress as the result of the aforementioned treatments and in the case of *M. minima* HC. Allelochemicals might be responsible for the alteration of water uptake in a direct (Einhellig, 1995b; Cheng and Cheng, 2015) or indirect way. In the second case, they can for example affect photosynthesis (Einhellig et al., 1993; Hussain and Reigosa, 2011): a decreased photosynthesis rate induces stomata closure which also results in a decreased water uptake (Cheng and Cheng, 2015).

For what concerns ROS, increasing evidence connects them to different stresses (Bogatek et al., 2007; Baxter et al., 2014). On the one hand, ROS have important roles in plant physiology, while on the other hand their imbalance results in disruption of cell ultrastructure and deregulation of many cellular processes (Mittler et al., 2004). A role of ROS in allelopathic interactions has been suggested (Bogatek et al., 2007): after exposure to allelochemicals, the receiving plant undergoes an alteration of antioxidant enzymes, in the attempt to counteract the induced redox imbalance (Gniazdowska et al., 2007). Nevertheless, ROS are often not sufficiently scavenged because they also can inactivate the scavenging enzymes (Oracz et al., 2007).

It has to be underlined that the effects related to water availability and redox imbalance, might be secondary effects and that time course experiments would be helpful in further shedding light on the primary responses. This information can then be used to design experiments aimed at identifying the mechanism of action and the molecular targets of the allelochemicals. Noteworthy, our results show once again that different stress phenomena seem to elicit a common set of responses (Kaplan et al., 2004).

### Identification of Putative Allelochemicals: The Whole is Greater Than the Sum of its Parts

The occurrence of metabolites in different fractions of the donor plants triggering similar responses in the receiving plant was used as hint to select candidate allelochemicals. The extracts of *A. unedo* and *M. communis* showed a very similar activity (**Figure 4**) and their metabolic profiles also showed significant similarities (**Tables 1** and **2**, **Figure 6**). Catechin and the flavonoids quercitrin and myricitrin were present in both plants but, since the differences in concentrations for the latter two were very high, catechin (detected at similar levels in the two extracts) was selected as candidate allelochemical. However, the growth-inhibiting activity of catechin (**Figure 7**) appeared to be weaker than that of the extracts and M fractions (**Figure 2**, **Table S1**). This suggests a significant contribution of other compounds. Further studies aimed at their identification are needed in order to prove this hypothesis.

Other metabolites chosen for testing their allelopathic activities based on the same considerations (occurrence in different fractions inducing similar responses) were arbutin, gallic, quinic and shikimic acid, all of which partitioned in the SPE water fractions. Quinic acid and shikimic acid, which both are ubiquitously occurring in plants, were found at high levels in the extracts of our donor plants. To the best of our knowledge, effects of these compounds on the root growth have been studied here for the first time, but no growth-inhibiting activity was found. Although gallic acid has been proven to induce ROS stress in Arabidopsis seedlings and to act on the microtubule assembly (Rudrappa et al., 2007), the allelopathic activity of the extracts of *A. geniculata* cannot be associated to this metabolite.

A discrete activity in the mM concentration was observed for arbutin, which was shown to be taken up by the receiving plant (**Figures 7**–**9**). However, the growth-inhibiting activity was not as strong as the one exerted by the whole extract (**Figures 2**–**4**). Arbutin has already been identified as possible allelochemical acting on soil microorganisms (Castaldi et al., 2009). In our study, its direct effects on a target plant have been shown for the first time, to the best of our knowledge. The results suggest arbutin as the main component responsible for the activity of *A. unedo* and *M. communis* SPE water fraction, although there are co-occurring compounds that may contribute to modulate and increase its activity.

In case of *M. minima*, it was not possible to narrow down the activity to single compounds, and instead it is very likely that compounds that separate in the two SPE fractions work synergistically to induce the drastic effects observed for the HC extract (**Figure 4A**, **Figures S7** and **S8**).

The occurrence of synergistic or additive effects in allelopathy has long been speculated (Blum, 1995; Einhellig, 1995a). Einhellig (1995a) suggested that the allelopathic inhibition under natural conditions is the result of the combined effect of several compounds.

This study confirmed that, although it is possible to identify single compounds as main active principles of a plant, the mixtures are much more active and trigger different and more drastic responses (**Figures 2**–**4**, **7**, and **8**, **Figure S12**). Different effects at metabolic level mean either different mechanisms of action or occurrence of interactions among different mechanisms induced by different molecules or finally mode of actions that require two or more components (a similar concept as adjuvants for vaccines).

The vital question is then how it is possible to detect synergism to occur in the field situation when it is already hard to explain how one chemical species can reach the receiving plant. The receiving plant can be reached at the same time by different compounds, not necessarily emitted by the same donor plant, that can enhance directly or indirectly the activity of the allelochemical in question. Or if we assume the adjuvant kind of mechanism, then the second component could be a rather ubiquitous compound. If this hypothesis could be confirmed, it would also have a significant impact on practical applications in herbicides formulation. Finally, although the concentrations used in the bioassays here discussed are in line with those generally used for phytotoxicity assessment of plant extracts (Scognamiglio et al., 2013), the determination of the concentrations of the active compounds in the field, as well as of their bioavailability, is a very important step to definitely validate the ecological significance of the observations herewith reported.

The results described here pave the way for extending studies on chemical interactions between plants. In particular, synergistic effects of allelochemicals need to be further explored both in controlled environment and in the field, without forgetting the crucial impact of microorganisms.

## Data Availability Statement

The raw data supporting the conclusions of this article will be made available by the authors, without undue reservation.

## Author Contributions

MS designed the project and the experiments with the supervision of BS. MS performed the experiments and data analyses. Both authors discussed the results and contributed to the final manuscript.

## Funding 

This work was supported by the Alexander von Humboldt Foundation through the fellowship granted to MS.

## Conflict of Interest

The authors declare that the research was conducted in the absence of any commercial or financial relationships that could be construed as a potential conflict of interest.
