# Supplementary material for: Identification of Potential Allelochemicals From Donor Plants and Their Synergistic Effects on the Metabolome of Aegilops geniculata
Source: Front Plant Sci. 2020 Aug 5;11:1046. doi: 10.3389/fpls.2020.01046 (PMC7419652; doi:10.3389/fpls.2020.01046)
Supplement: Supplementary file 1 [file DataSheet_1.docx]

**Identification of potential allelochemicals from donor plants and their synergistic effects on the metabolome of *Aegilops geniculata***

Monica Scognamiglio^[[1]](#footnote-1)^*, Bernd Schneider

Max Planck Institute for Chemical Ecology, Hans-Knöll-Str. 8, 07745 Jena, Germany

**Supplementary Material**

**Figure S1:** Pictures of *Aegilops geniculata*: A) a control plant; B,C,D) plants treated with *Arbutus unedo*, *Myrtus communis* and *Medicago minima*, respectively.

B

A

D

C

**Figure S2:** Principal component analysis of ^1^H NMR data of samples obtained from the receiving plant, *A. geniculata*, treated with the donor plant extracts and SPE fractions: loading column plots.

A/B= loading plots of pc1 and pc2, respectively of leaf samples of *A. geniculata* treated with the three extracts at two different concentrations;

C/D= loading plots of pc1 and pc2, respectively of root samples of *A. geniculata* treated with the three extracts at two different concentrations;

E/F= loading plots of pc1 and pc2, respectively of leaf samples of *A. geniculata* treated with *A. unedo* extracts and SPE fractions;

G/H= loading plots of pc1 and pc2, respectively of root samples of *A. geniculata* treated with *A. unedo* extracts and SPE fractions;

I/J= loading plots of pc1 and pc2, respectively of leaf samples of *A. geniculata* treated with *M. communis* extracts and SPE fractions;

K/L= loading plots of pc1 and pc2, respectively of root samples of *A. geniculata* treated with *M. communis* extracts and SPE fractions;

M/N= loading plots of pc1 and pc2, respectively of leaf samples of *A. geniculata* treated with *M. minima* extracts and SPE fractions;

O/P= loading plots of pc1 and pc2, respectively of root samples of *A. geniculata* treated with *M. minima* extracts and SPE fractions.

A

B

C


D

F

E

G

H

I

J

K

L

M

N

O

P

**Figure S3:** Oblongaroside A

**Figure S4:** PLS DA A) score and B) loading plots of leaf samples from the receiving plant, *A. geniculata*, treated with the donor plant extracts and SPE fractions. Circles indicate the control and the groups where few metabolic changes were observed, asterisk groups composed of the plants treated with the active extracts and fractions.

A

B

**Figure S5:** PLS DA A) score and B) loading plots of root samples from the receiving plant, *A. geniculata*, treated with the donor plant extracts and SPE fractions. Circles indicate the control and the groups where few metabolic changes were observed, asterisk groups composed of the plants treated with the active extracts and fractions.

A

B

**Figure S6:** Pathway analysis obtained using the relative function in Metaboanalyst.

**
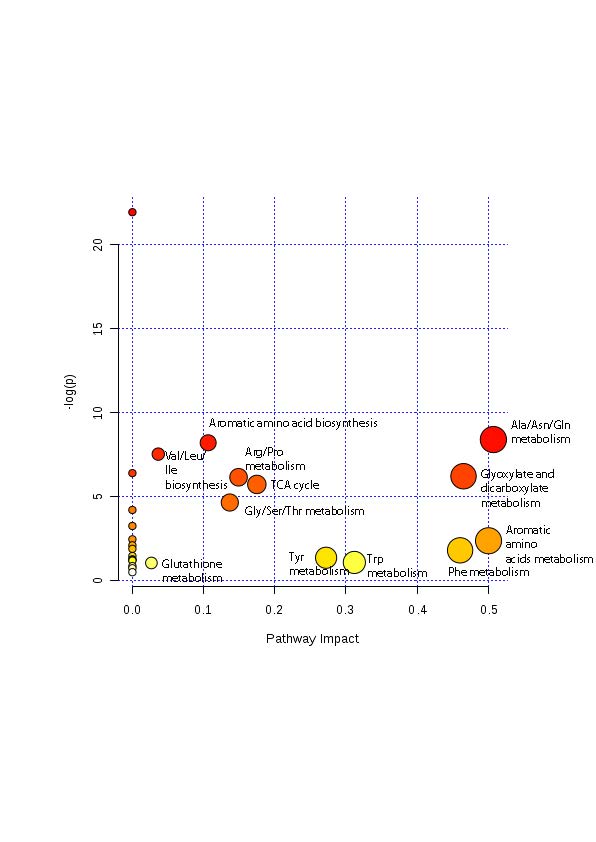
**

**Figure S7:** NMR spectra (700 MHz, MeOH-*d*_4_: phosphate buffer in D_2_O 1:1) of leaf of *A. geniculata*. Controls: black; HC=dark green; LC=light green; Methanol SPME fraction= red; Water SPME fraction= blue. The region around 3.5-4.0 ppm is not shown.

**Figure S8:** NMR spectra (700 MHz, MeOH-*d*_4_ : phosphate buffer in D_2_O 1:1) of root of *A. geniculata*. Controls: black; HC=dark green; LC=light green; Methanol SPME fraction= red; Water SPME fraction= blue. The region around 3.5-4.0 ppm is not shown.

**Table S1:** Morphological analysis of *Aegilops geniculata* plants treated with donor plant extracts: % variation from control of root elongation (±SD; n=10) on data normalized against 7 days old root length. H, M, L= high, medium, low concentration, respectively; SPEM, SPEW= SPE methanol and water fractions, respectively.

| **Donor plant species** | **Samples** | **Variation from control** |
| --- | --- | --- |
| ***Arbutus unedo*** | H | -68% ± 77% |
|  | M | -167% ± 68% |
|  | L | -89% ± 42% |
|  | SPEM | 43% ± 213% |
|  | SPEW | -17% ± 200% |
| ***Myrtus communis*** | H | 61% ± 84% |
|  | M | -162% ± 79% |
|  | L | -147% ± 61% |
|  | SPEM | 221% ± 415% |
|  | SPEW | 368% ± 388% |
| ***Medicago minima*** | H | -321% ± 349% |
|  | M | -432% ± 472% |
|  | L | -64% ± 314% |
|  | SPEM | 223% ± 527% |
|  | SPEW | -37% ± 223% |

**Table S2:** Top 50 features identified by One-way ANOVA and post-hoc analysis of data obtained from the analysis of *A. geniculata* leaf samples. The analyses were carried out with Metaboanalyst 4.0.

|  | **NMR Bucket (ppm)** | **Compound** | **F value** | **P value** | **-log10(p)** | **FDR*** |
| --- | --- | --- | --- | --- | --- | --- |
| 1 | 3.54 - 3.58 | cis-Aconitate | 41.165 | 1.26E-23 | 22.898 | 2.34E-21 |
| 2 | 7.14 - 7.18 | Trp/Tyr | 31.514 | 9.06E-21 | 20.043 | 8.38E-19 |
| 3 | 7.30 - 7.34 | Phe | 30.814 | 1.56E-20 | 19.808 | 9.60E-19 |
| 4 | 2.14 - 2.18 | Glu/Shikimate | 26.277 | 6.82E-19 | 18.166 | 3.16E-17 |
| 5 | 6.46 - 6.50 | Shikimate | 25.053 | 2.07E-18 | 17.683 | 7.67E-17 |
| 6 | 3.26 - 3.30 | Betaine | 24.291 | 4.23E-18 | 17.373 | 1.31E-16 |
| 7 | 7.18 - 7.22 | Trp/Tyr | 23.197 | 1.22E-17 | 16.914 | 3.22E-16 |
| 8 | 7.26 - 7.30 | Trp | 22.064 | 3.81E-17 | 16.42 | 8.77E-16 |
| 9 | 2.42 - 2.46 | Glu/Malate | 21.953 | 4.27E-17 | 16.37 | 8.77E-16 |
| 10 | 7.38 - 7.42 | Phe | 21.301 | 8.41E-17 | 16.075 | 1.56E-15 |
| 11 | 2.10 - 2.14 | Glu | 19.632 | 5.15E-16 | 15.288 | 8.66E-15 |
| 12 | 6.78 - 6.82 | Arbutin/ Phenolic compound | 18.336 | 2.29E-15 | 14.641 | 3.53E-14 |
| 13 | 7.34 - 7.38 | Phe | 17.826 | 4.21E-15 | 14.376 | 5.82E-14 |
| 14 | 5.02 - 5.06 | Oblongaroside A | 17.788 | 4.41E-15 | 14.356 | 5.82E-14 |
| 15 | 6.86 - 6.90 | Phenolic compound | 17.563 | 5.78E-15 | 14.238 | 7.13E-14 |
| 16 | 1.74 - 1.78 | Oblongaroside A | 17.497 | 6.27E-15 | 14.203 | 7.25E-14 |
| 17 | 4.94 - 4.98 | Oblongaroside A | 16.332 | 2.70E-14 | 13.568 | 2.94E-13 |
| 18 | 1.94 - 1.98 | Pro | 14.914 | 1.78E-13 | 12.751 | 1.83E-12 |
| 19 | 1.98 - 2.02 | Pro | 14.385 | 3.70E-13 | 12.431 | 3.61E-12 |
| 20 | 1.02 - 1.06 | Val | 14.165 | 5.06E-13 | 12.296 | 4.68E-12 |
| 21 | 2.46 - 2.50 | Glu der/Glu/Malate | 13.694 | 9.96E-13 | 12.002 | 8.77E-12 |
| 22 | 2.02 - 2.06 | Pro/Glu der/Gln/Quinate | 13.66 | 1.05E-12 | 11.98 | 8.81E-12 |
| 23 | 6.82 - 6.86 | Tyr | 13.25 | 1.92E-12 | 11.718 | 1.54E-11 |
| 24 | 2.06 - 2.10 | Gln/Pro | 13.102 | 2.39E-12 | 11.622 | 1.84E-11 |
| 25 | 2.34 - 2.38 | Pro | 11.705 | 2.10E-11 | 10.678 | 1.55E-10 |
| 26 | 3.86 - 3.90 | Betaine | 11.563 | 2.65E-11 | 10.578 | 1.88E-10 |
| 27 | 2.94 - 2.98 | Asn | 11.226 | 4.60E-11 | 10.338 | 3.15E-10 |
| 28 | 1.10 - 1.14 | Val der | 10.824 | 9.02E-11 | 10.045 | 5.96E-10 |
| 29 | 7.22 - 7.26 | Trp | 9.9269 | 4.30E-10 | 9.3662 | 2.75E-09 |
| 30 | 0.94 - 0.98 | Leu/Ile | 9.8892 | 4.60E-10 | 9.3369 | 2.84E-09 |
| 31 | 0.98 - 1.02 | Val | 9.424 | 1.07E-09 | 8.9699 | 6.40E-09 |
| 32 | 7.70 - 7.74 | Trp | 9.3017 | 1.34E-09 | 8.8717 | 7.77E-09 |
| 33 | 7.02 - 7.06 | Arbutin/ Phenolic compound | 9.2669 | 1.43E-09 | 8.8436 | 8.04E-09 |
| 34 | 1.66 - 1.70 | Amino acids (overlapped) | 9.1706 | 1.72E-09 | 8.7656 | 9.22E-09 |
| 35 | 6.94 - 6.98 | Arbutin/ Phenolic comp | 9.162 | 1.74E-09 | 8.7586 | 9.22E-09 |
| 36 | 1.06 - 1.10 | Val | 9.082 | 2.03E-09 | 8.6933 | 1.04E-08 |
| 37 | 7.10 - 7.14 | Trp | 8.5847 | 5.24E-09 | 8.2806 | 2.58E-08 |
| 38 | 4.06 - 4.10 | Sugars/ amino acids (overlapped) | 8.5681 | 5.41E-09 | 8.2666 | 2.58E-08 |
| 39 | 2.82 - 2.86 | Asn | 8.5647 | 5.45E-09 | 8.2637 | 2.58E-08 |
| 40 | 4.30 - 4.34 | Oblongaroside A | 7.3689 | 6.11E-08 | 7.2142 | 2.82E-07 |
| 41 | 6.98 - 7.02 | Arbutin/ Phenolic comp | 7.2095 | 8.55E-08 | 7.0679 | 3.86E-07 |
| 42 | 2.70 - 2.74 | Citrate/Malate | 7.1597 | 9.51E-08 | 7.0219 | 4.19E-07 |
| 43 | 4.18 - 4.22 | Thr | 6.4586 | 4.39E-07 | 6.3574 | 1.89E-06 |
| 44 | 4.22 - 4.26 | Thr | 6.4149 | 4.84E-07 | 6.3149 | 2.04E-06 |
| 45 | 7.06 - 7.10 | Phenolic compound | 6.3768 | 5.28E-07 | 6.2777 | 2.16E-06 |
| 46 | 1.90 - 1.94 | GABA/Quinate | 6.3692 | 5.37E-07 | 6.2703 | 2.16E-06 |
| 47 | 3.66 - 3.70 | Sugars/aa ov | 6.348 | 5.63E-07 | 6.2496 | 2.22E-06 |
| 48 | 2.54 - 2.58 | Citrate | 6.2617 | 6.84E-07 | 6.1649 | 2.64E-06 |
| 49 | 2.86 - 2.90 | Asn derivative | 6.2183 | 7.55E-07 | 6.1221 | 2.85E-06 |
| 50 | 6.66 - 6.70 | cis-Aconitate | 6.2101 | 7.69E-07 | 6.1141 | 2.85E-06 |

*FDR= False discovery rate

**Table S3:** Top 50 features identified by One-way ANOVA and post-hoc analysis of data obtained from the analysis of *A.* *geniculata* root samples. The analyses were carried out with Metaboanalyst 4.0.

|  | **NMR Bucket**  **(ppm)** | **Compound** | **F value** | **P value** | **-log10(p)** | **FDR*** |
| --- | --- | --- | --- | --- | --- | --- |
| 1 | 6.86 - 6.90 | Phenolic compound | 36.29 | 9.10E-22 | 21.041 | 1.68E-19 |
| 2 | 6.74 - 6.78 | Phenolic compound | 32.057 | 1.72E-20 | 19.766 | 1.59E-18 |
| 3 | 6.46 - 6.50 | Phenolic compound | 31.191 | 3.26E-20 | 19.487 | 1.85E-18 |
| 4 | 7.42 - 7.46 | Phenolic compound | 30.921 | 3.99E-20 | 19.399 | 1.85E-18 |
| 5 | 6.82 - 6.86 | Phenolic compound | 28.083 | 3.71E-19 | 18.431 | 1.37E-17 |
| 6 | 6.30 - 6.34 | Flavonoids | 26.243 | 1.74E-18 | 17.759 | 5.37E-17 |
| 7 | 6.66 - 6.70 | cis-Aconitate | 23.826 | 1.53E-17 | 16.815 | 4.05E-16 |
| 8 | 6.90 - 6.94 | Phen compound/Gallic acid derivative | 23.438 | 2.21E-17 | 16.656 | 4.92E-16 |
| 9 | 3.58 - 3.62 | Sugars (overlapped) | 23.353 | 2.39E-17 | 16.621 | 4.92E-16 |
| 10 | 6.58 - 6.62 | Shikimic acid | 22.333 | 6.42E-17 | 16.193 | 1.19E-15 |
| 11 | 6.98 - 7.02 | Phenolic compound/ Gallic acid derivative | 21.887 | 1.00E-16 | 16 | 1.68E-15 |
| 12 | 6.62 - 6.66 | Phenolic compound | 21.679 | 1.23E-16 | 15.909 | 1.90E-15 |
| 13 | 7.06 - 7.10 | Phenolic compound/ Gallic acid derivative | 21.532 | 1.43E-16 | 15.845 | 1.90E-15 |
| 14 | 5.26 - 5.30 | Rha | 21.526 | 1.44E-16 | 15.842 | 1.90E-15 |
| 15 | 1.14 - 1.18 | Amino acids (overlapped) | 21.202 | 2.00E-16 | 15.699 | 2.47E-15 |
| 16 | 6.22 - 6.26 | Phenolic compound | 20.948 | 2.60E-16 | 15.585 | 3.01E-15 |
| 17 | 1.94 - 1.98 | Acetate | 19.719 | 9.58E-16 | 15.019 | 1.04E-14 |
| 18 | 6.94 - 6.98 | Phenolic compound | 19.134 | 1.82E-15 | 14.74 | 1.87E-14 |
| 19 | 6.78 - 6.82 | Phenolic compound | 18.97 | 2.19E-15 | 14.66 | 2.13E-14 |
| 20 | 6.38 - 6.42 | Phenolic compound | 17.351 | 1.42E-14 | 13.847 | 1.31E-13 |
| 21 | 7.26 - 7.30 | Phenolic compound | 17.229 | 1.65E-14 | 13.784 | 1.45E-13 |
| 22 | 6.26 - 6.30 | Phenolic compound | 17.045 | 2.06E-14 | 13.687 | 1.73E-13 |
| 23 | 6.34 - 6.38 | Phenolic compound | 16.947 | 2.31E-14 | 13.635 | 1.86E-13 |
| 24 | 6.50 - 6.54 | Flavonoids | 16.868 | 2.55E-14 | 13.593 | 1.97E-13 |
| 25 | 3.54 - 3.58 | Sugars (overlapped) | 16.57 | 3.68E-14 | 13.435 | 2.72E-13 |
| 26 | 7.02 - 7.06 | Phenolic compound/ Gallic acid derivative | 16.252 | 5.47E-14 | 13.262 | 3.89E-13 |
| 27 | 6.42 - 6.46 | Phenolic compound | 16.034 | 7.19E-14 | 13.143 | 4.93E-13 |
| 28 | 0.90 - 0.94 | Rha | 15.979 | 7.72E-14 | 13.113 | 5.10E-13 |
| 29 | 2.54 - 2.58 | Citrate | 15.581 | 1.29E-13 | 12.891 | 8.20E-13 |
| 30 | 6.70 - 6.74 | Phenolic compound | 15.148 | 2.26E-13 | 12.645 | 1.40E-12 |
| 31 | 7.10 - 7.14 | Phenolic compound/ Gallic acid derivative | 15.062 | 2.54E-13 | 12.596 | 1.51E-12 |
| 32 | 6.54 - 6.58 | Shikimic acid | 15.037 | 2.62E-13 | 12.581 | 1.52E-12 |
| 33 | 1.06 - 1.10 | Val | 14.85 | 3.36E-13 | 12.473 | 1.89E-12 |
| 34 | 7.22 - 7.26 | Phenolic compound | 14.711 | 4.06E-13 | 12.392 | 2.21E-12 |
| 35 | 1.18 - 1.22 | Val | 14.223 | 7.90E-13 | 12.103 | 4.17E-12 |
| 36 | 7.14 - 7.18 | Phenolic compound/ Gallic acid derivative | 14.108 | 9.26E-13 | 12.033 | 4.76E-12 |
| 37 | 2.42 - 2.46 | Glu/Malate | 13.904 | 1.23E-12 | 11.91 | 6.09E-12 |
| 38 | 2.82 - 2.86 | Asn | 13.893 | 1.25E-12 | 11.903 | 6.09E-12 |
| 39 | 4.02 - 4.06 | Gln | 13.584 | 1.93E-12 | 11.714 | 9.17E-12 |
| 40 | 2.94 - 2.98 | Asn | 13.518 | 2.13E-12 | 11.672 | 9.84E-12 |
| 41 | 2.38 - 2.42 | Malate | 12.896 | 5.25E-12 | 11.279 | 2.37E-11 |
| 42 | 2.22 - 2.26 | Amino acids (overlapped) | 12.781 | 6.23E-12 | 11.206 | 2.74E-11 |
| 43 | 7.34 - 7.38 | Phenolic compound | 12.609 | 8.04E-12 | 11.095 | 3.46E-11 |
| 44 | 3.66 - 3.70 | Pro | 12.455 | 1.01E-11 | 10.994 | 4.27E-11 |
| 45 | 1.98 - 2.02 | Pro | 12.391 | 1.12E-11 | 10.951 | 4.60E-11 |
| 46 | 5.30 - 5.34 | Sugars ov | 12.279 | 1.33E-11 | 10.878 | 5.33E-11 |
| 47 | 5.22 - 5.26 | Unknown | 11.986 | 2.08E-11 | 10.682 | 8.18E-11 |
| 48 | 5.14 - 5.18 | Glc | 11.88 | 2.45E-11 | 10.61 | 9.46E-11 |
| 49 | 7.30 - 7.34 | Phenolic compound | 11.767 | 2.93E-11 | 10.534 | 1.10E-10 |
| 50 | 5.90 - 5.94 | Unknown | 11.534 | 4.23E-11 | 10.374 | 1.56E-10 |

*FDR= False discovery rate

**Table S4:** Confidence level of metabolite identification.

| **Confidence level** | **Definition** | **Explanation** |
| --- | --- | --- |
| 1 | Unequivocally identified compound | Complete characterization through 1 and 2D NMR (attribution of all of the signals and all of the crucial homo and hetero-correlations) |
| 2 | Putatively identified compound | Not all the signals or correlations were detected in the extracts |
| 3 | Putatively identified compound class | Based on the NMR data, it was possible to identify the class of the compound but either a portion of the molecule was not identified, or some crucial correlations were missing to univocally identify the compound |
| 4 | Unknown compound | It was not possible to propose a structure for the detected signals or, even in case of proposed structure, there was not sufficient supporting data |

**Table S5:** Database and main literature sources used for metabolomics data.

| **Databases** | **Reference** |
| --- | --- |
| Madison Metabolomics Consortium Database | Cui et al., 2008 |
| Human Metabolome Database | Wishart et al., 2018 |
| Pubchem | https://www.ncbi.nlm.nih.gov/pccompound |
| **Literature** | |
| Lubbe et al., 2013 | |
| Scognamiglio et al., 2014 | |
| Scognamiglio et al., 2014 | |
| Verpoorte et al., 2007 | |

**Table S6:** Literature supporting the structural elucidation of compounds carried out by 2D-NMR data and used NMR solvents (as reported in Table 2).

| **Compound** | **References for presence of the compound in the studied species or in species of the same genus/ family** | **References for NMR data of compounds identified with confidence levels 1 and 2**  **(used NMR solvent)** |
| --- | --- | --- |
| Arbutin | Fiorentino et al., 2007  Xu et al., 2015 | Kim et al., 2010  (MeOH-d*_4_*: phosphate buffer in D_2_O 1:1, pH 6.0)  Scognamiglio et al., 2014  (MeOH-d*_4_*: phosphate buffer in D_2_O 1:1, pH 6.0) |
| Gallic acid | Pawlowska et al., 2006  Romani et al., 2012 | Lee et al., 2011  (deionized water containing 90% D_2_O, pH 5.5)  Scognamiglio et al., 2014  (MeOH-d*_4_*: phosphate buffer in D_2_O 1:1, pH 6) |
| Galloylarbutin | Miguel et al., 2014  Xu et al., 2015 |  |
| Glucogallin | Miguel et al., 2014 | Puppala et al., 2012  (Acetone-d*_6_* and D_2_O) |
| Ellagic acid | Taamalli et al., 2014 | Li et al., 1999  (DMSO-d*_6_*) |
| Quinic acid | Miguel et al., 2014  Diaz-de-Cerio et al., 2018 | Scognamiglio et al., 2014  (MeOH-d*_4_*: phosphate buffer in D_2_O 1:1, pH 6) |
| 5-galloyl shikimic acid | Mendes et al., 2011  Diaz-de-Cerio et al., 2018 |  |
| Shikimic acid | Avula et al., 2009 | Scognamiglio et al., 2014  (MeOH-d*_4_*: phosphate buffer in D_2_O 1:1, pH 6) |
| Catechin | Miguel et al., 2014  Romani et al., 1999 | Wolfender et al., 2013  (MeOH-d*_4_*: phosphate buffer in D_2_O 1:1, pH 6) |
| Luteolin derivatives | Stochmal et al., 2001 | Wolfender et al., 2013  (MeOH-d*_4_*: phosphate buffer in D_2_O 1:1, pH 6) |
| Myricetin | Romani et al., 1999 | Wolfender et al., 2013  (MeOH-d*_4_*: phosphate buffer in D_2_O 1:1, pH 6) |
| Myricitrin | Miguel et al., 2014  Romani et al., 1999 | Hwang et al., 2018  (MeOH-d*_4_*) |
| Quercitrin | Miguel et al., 2014  Romani et al., 1999 | Wolfender et al., 2013  (MeOH-d*_4_*: phosphate buffer in D_2_O 1:1, pH 6) |
| Rutin | Malheiro et al., 2012 | Scognamiglio et al., 2014  (MeOH-d*_4_*: phosphate buffer in D_2_O 1:1, pH 6) |
| Daidzein | Rodrigues et al., 2014 |  |
| Daidzin | Rodrigues et al., 2014 |  |
| Genistein | Rodrigues et al., 2014 |  |
| Monotropein | Karikas et al., 1987 | Tzakou et al., 2007  (DMSO-d_6_) |
| Oleoside 11-methyl ester |  | Dinda, 2019  (MeOH-d*_4_*) |
| Unedoside | Karikas et al., 1987 | Jensen et al., 1998  (D_2_O) |
| Trigonelline | Naidu et al., 1992 | Lubbe et al., 2013  (MeOH-d*_4_*: phosphate buffer in D_2_O 1:1, pH 6) |
| Myrtucommulone | Celaj et al., 2020 |  |

**Methods S1:** Approach to the identification of compounds.

Compound identity was confirmed by extensive 2D NMR analysis as reported in Table 2. Besides the 2D correlations reported there, TOCSY and/or HSQC-TOCSY were used to confirm the spin systems. When possible, data were compared with spectra of pure compounds (this was done for the biologically relevant metabolites), with the literature and with data in databases (references reported in Tables S5 and S6).

The confidence level for the identification of each metabolite is indicated by a number (1-4, see table S4 for explanation) reported in parentheses after the compound name, in accordance with the directions for the minimum reporting standards in metabolomics (Sumner et al., 2007).

**References SM**

Avula, B., Wang, Y.H., Smillie, T.J., and Khan, I. (2009). Determination of Shikimic Acid in Fruits of *Illicium* Species and Various Other Plant Samples by LC-UV and LC-ESI-MS. *Chromatographia* 69**,** 307-314.

Celaj, O., Durán, A. G., Cennamo, P., Scognamiglio, M., Fiorentino, A., Esposito, A., & D’Abrosca, B. (2020). Phloroglucinols from Myrtaceae: attractive targets for structural characterization, biological properties and synthetic procedures. *Phytochemistry Reviews*, 1-41. <https://doi.org/10.1007/s11101-020-09697-2>”

Cui, Q., Lewis, I.A., Hegeman, A.D., Anderson, M.E., Li, J., Schulte, C.F., Westler, W.M., Eghbalnia, H.R., Sussman, M.R., and Markley, J.L. (2008). Metabolite identification via the Madison Metabolomics Consortium Database. *Nature Biotechnology* 26**,** 162-164.

Diaz-De-Cerio, E., Arraez-Roman, D., Segura-Carretero, A., Ferranti, P., Nicoletti, R., Perrotta, G.M., and Gomez-Caravaca, A.M. (2018). Establishment of pressurized-liquid extraction by response surface methodology approach coupled to HPLC-DAD-TOF-MS for the determination of phenolic compounds of myrtle leaves. *Analytical and Bioanalytical Chemistry* 410**,** 3547-3557.

Dinda, Biswanath (2019). *Pharmacology and Applications of Naturally Occurring Iridoids*. Springer International Publishing.

Fiorentino, A., Castaldi, S., D'abrosca, B., Natale, A., Carfora, A., Messere, A., and Monaco, P. (2007). Polyphenols from the hydroalcoholic extract of *Arbutus unedo* living in a monospecific Mediterranean woodland. *Biochemical Systematics and Ecology* 35**,** 809-811.

Hwang, In-Wook, and Shin-Kyo Chung (2018). Isolation and identification of myricitrin, an antioxidant flavonoid, from daebong persimmon peel. *Preventive nutrition and food science* 23, 341.

Jensen, S.R., Ravnkilde, L., and Schripsema, J. (1998). Unedoside derivatives in *Nuxia* and their biosynthesis. *Phytochemistry* 47**,** 1007-1011.

Karikas, George A., Melvin R. Euerby, and Roger D. Waighc (1987). "Constituents of the stems of *Arbutus unedo*." *Planta medica* 53, 223-224.

Kim, H.K., Saifullah, Khan, S., Wilson, E.G., Kricun, S.D.P., Meissner, A., Goraler, S., Deelder, A.M., Choi, Y.H., and Verpoorte, R. (2010). Metabolic classification of South American Ilex species by NMR-based metabolomics. *Phytochemistry* 71**,** 773-784.

Lee, J.E., Lee, B.J., Chung, J.O., Shin, H.J., Lee, S.J., Lee, C.H., and Hong, Y.S. (2011). H-1 NMR-based metabolomic characterization during green tea (*Camellia sinensis*) fermentation. *Food Research International* 44**,** 597-604.

Li, X.C., Elsohly, H.N., Hufford, C.D., and Clark, A.M. (1999). NMR assignments of ellagic acid derivatives. *Magnetic Resonance in Chemistry* 37**,** 856-859.

Lubbe, A., Gude, H., Verpoorte, R., and Choi, Y.H. (2013). Seasonal accumulation of major alkaloids in organs of pharmaceutical crop *Narcissus* Carlton. *Phytochemistry* 88**,** 43-53.

Malheiro, R., Sa, O., Pereira, E., Aguiar, C., Baptista, P., and Pereira, J.A. (2012). *Arbutus unedo* L. leaves as source of phytochemicals with bioactive properties. *Industrial Crops and Products* 37**,** 473-478.

Mendes, L., De Freitas, V., Baptista, P., and Carvalho, M. (2011). Comparative antihemolytic and radical scavenging activities of strawberry tree (*Arbutus unedo* L.) leaf and fruit. *Food and Chemical Toxicology* 49**,** 2285-2291.

Miguel, M.G., Faleiro, M.L., Guerreiro, A.C., and Antunes, M.D. (2014). *Arbutus unedo* L.: Chemical and Biological Properties. *Molecules* 19**,** 15799-15823.

Naidu, B.P., Paleg, L.G., and Jones, G.P. (1992). Nitrogenous Compatible Solutes in Drought-Stressed *Medicago* Spp. *Phytochemistry* 31**,** 1195-1197.

Pawlowska, A.M., De Leo, M., and Braca, A. (2006). Phenolics of Arbutus unedo L. (Ericaceae) fruits: Identification of anthocyanins and gallic acid derivatives. *Journal of Agricultural and Food Chemistry* 54**,** 10234-10238.

Puppala, M., Ponder, J., Suryanarayana, P., Reddy, G.B., Petrash, J.M., and Labarbera, D.V. (2012). The Isolation and Characterization of beta-Glucogallin as a Novel Aldose Reductase Inhibitor from Emblica officinalis. *Plos One* 7.

Rodrigues, F., Almeida, I., Sarmento, B., Amaral, M.H., and Oliveira, M.B.P.P. (2014). Study of the isoflavone content of different extracts of *Medicago* spp. as potential active ingredient. *Industrial Crops and Products* 57**,** 110-115.

Romani, A., Campo, M., and Pinelli, P. (2012). HPLC/DAD/ESI-MS analyses and anti-radical activity of hydrolyzable tannins from different vegetal species. *Food Chemistry* 130**,** 214-221.

Romani, A., Pinelli, P., Mulinacci, N., Vincieri, F.F., and Tattini, M. (1999). Identification and quantitation of polyphenols in leaves of *Myrtus communis* L. *Chromatographia* 49**,** 17-20.

Scognamiglio, M., Fiumano, V., D'abrosca, B., Esposito, A., Choi, Y.H., Verpoorte, R., and Fiorentino, A. (2014). Chemical interactions between plants in Mediterranean vegetation: The influence of selected plant extracts on *Aegilops geniculata* metabolome. *Phytochemistry* 106**,** 69-85.

Scognamiglio, M., D’Abrosca, B., Esposito, A., & Fiorentino, A. (2015). Chemical composition and seasonality of aromatic mediterranean plant species by NMR-based metabolomics. *Journal of analytical methods in chemistry*, *2015*.

Stochmal, A., Piacente, S., Pizza, C., De Riccardis, F., Leitz, R., and Oleszek, W. (2001). Alfalfa (*Medicago sativa* L.) flavonoids. 1. Apigenin and luteolin glycosides from aerial parts. *Journal of Agricultural and Food Chemistry* 49**,** 753-758.

Sumner, L. W., Amberg, A., Barrett, D., Beale, M. H., Beger, R., Daykin, C. A., ... & Hankemeier, T. (2007). Proposed minimum reporting standards for chemical analysis. *Metabolomics*, *3*(3), 211-221.

Taamalli, A., Iswaldi, I., Arraez-Roman, D., Segura-Carretero, A., Fernandez-Gutierrez, A., and Zarrouk, M. (2014). UPLC-QTOF/MS for a Rapid Characterisation of Phenolic Compounds from Leaves of *Myrtus communis* L. *Phytochemical Analysis* 25**,** 89-96.

Tzakou, O., Mylonas, P., Vagias, C., and Petrakis, P.V. (2007). Iridoid glucosides with insecticidal activity from *Galium melanantherum*. *Zeitschrift Fur Naturforschung C-a Journal of Biosciences* 62**,** 597-602.

Verpoorte, R., Choi, Y. H., & Kim, H. K. (2007). NMR-based metabolomics at work in phytochemistry. *Phytochemistry reviews*, *6*(1), 3-14.

Wishart, D.S., Feunang, Y.D., Marcu, A., Guo, A.C., Liang, K., Vazquez-Fresno, R., Sajed, T., Johnson, D., Li, C.R., Karu, N., Sayeeda, Z., Lo, E., Assempour, N., Berjanskii, M., Singhal, S., Arndt, D., Liang, Y.J., Badran, H., Grant, J., Serra-Cayuela, A., Liu, Y.F., Mandal, R., Neveu, V., Pon, A., Knox, C., Wilson, M., Manach, C., and Scalbert, A. (2018). HMDB 4.0: the human metabolome database for 2018. *Nucleic Acids Research* 46**,** D608-D617.

Wolfender, J.L., Rudaz, S., Choi, Y.H., and Kim, H.K. (2013). Plant Metabolomics: From Holistic Data to Relevant Biomarkers. *Current Medicinal Chemistry* 20**,** 1056-1090.

Xu, W.H., Liang, Q., Zhang, Y.J., and Zhao, P. (2015). Naturally Occurring Arbutin Derivatives and Their Bioactivities. *Chemistry & Biodiversity* 12**,** 54-81.

1. * Corresponding author. E-mail: mscognamiglio@ice.mpg.de

   Current address: University of Campania “Luigi Vanvitelli”, Department of Environmental Biological and Pharmaceutical Sciences and Technologies. Tel: +39 (0)0823 274576; e-mail: monica.scognamiglio@unicampania.it [↑](#footnote-ref-1)
